# Bibliometric Analysis of Studies on Neuropathic Pain Associated With Depression or Anxiety Published From 2000 to 2020

**DOI:** 10.3389/fnhum.2021.729587

**Published:** 2021-09-06

**Authors:** Kai-Li-Mi Li, Yu-Meng Chen, Xue-Qiang Wang, Hao-Yu Hu

**Affiliations:** ^1^School of Kinesiology, Shanghai University of Sport, Shanghai, China; ^2^Department of Sport Rehabilitation, Shanghai University of Sport, Shanghai, China; ^3^Shanghai Shangti Orthopaedic Hospital, Shanghai, China

**Keywords:** neuropathic pain, depression, anxiety, bibliometrics, citespace, web of science

## Abstract

**Objective:** Neuropathic pain (NP) associated with depression or anxiety is highly prevalent in clinical practice. Publications about NP associated with depression or anxiety increased exponentially from 2000 to 2020. However, studies that applied the bibliometric method in analyzing global scientific research about NP associated with depression or anxiety are rare. This work used the bibliometric method to analyze the publications on NP associated with depression or anxiety between 2000 and 2020.

**Method:** Publications from 2000 and 2020 were identified from the Thomson Reuters Web of Science (WoS) database. We employed CiteSpace V to conduct the bibliometric study.

**Results:** A total of 915 articles or reviews were obtained from the WoS database. The number of publications has increased over the last two decades. The USA was the most productive among countries or regions in the field. According to the burst key words, neuroinflammation, hippocampus, safety, and modulation were the hot global research issues in the domain.

**Conclusion:** Publications about NP associated with depression or anxiety have remarkably increased from 2000 to 2020. These historical opinions about NP associated with depression or anxiety could be an important practical basis for further research into potential development trends.

## Introduction

Neuropathic pain (NP) is associated with various conditions involved in a lesion or disease of the somatosensory nervous system (Jensen and Finnerup, 2014). The main symptoms of NP include: (i) spontaneous pain, such as burning, shooting, pricking, and pin-and-needle sensations; (ii) allodynia, pain due to a stimulus that normally does not induce pain; and (iii) hyperalgesia, increased pain from a normally painful stimulus (Cavalli et al., 2019; Finnerup et al., 2021). Most patients with NP complain of continuous or occasionally spontaneous pain, which may occur with evoked pain (Baron et al., 2009; Baskozos et al., 2019; Finnerup et al., 2021). Allodynia and hyperalgesia commonly appear in a number of peripheral neuropathies and central pain disorders and affect 15%–50% of patients with NP (Jensen and Finnerup, 2014). Moreover, NP became a common health problem in different chronic diseases given its complex etiology and created a considerable medical burden worldwide (Cohen and Mao, 2014; Makris et al., 2014; van Hecke et al., 2014; Qaseem et al., 2017). Approximately 5% of the general population suffer from NP (Attal et al., 2011; Bannister et al., 2020), and the prevalence in the general European population is estimated to be between 6.9% and 10% (van Hecke et al., 2014; Bernetti et al., 2021). Moreover, quality-of-life studies reported that NP is a comorbidity of depression, poor sleep quality, and physical dysfunction (Gilron et al., 2015). In accordance with this expectation, a number of studies have focused on NP associated with mood disorders, such as depression and anxiety (Humo et al., 2019; Wang et al., 2019; Wei et al., 2020; Kremer et al., 2021).

Pain intensity is caused by an instance of growing levels of mood symptoms, such as depression and anxiety (Barman et al., 2015). The neurobiological mechanisms of pain and mood disorders are still incompletely understood. Results of neuroimaging field studies showed that depression can rebuild various brain areas related to pain processing and recognition (Bar et al., 2007; Barman et al., 2015; Wang et al., 2019). Previous studies indicated that the midbrain dopamine circuit is an important modulator of pain-induced anxiety or depression symptoms (Mitsi and Zachariou, 2016). In addition, depressive disorders are classified from dysthymia to depressive episodes with psychotic characteristics (Michaelides and Zis, 2019). Epidemiologic studies in the field concluded that patients who suffer from a major depressive disorder are six times more likely to suffer from NP (Jackson et al., 2014; Zis et al., 2017; Michaelides and Zis, 2019). Generalized anxiety is the most common mental disorder, and about 20% of adults suffer from anxiety disorders every year (Munir and Takov, 2021). An extraordinarily high prevalence of depression and anxiety disorders occurs among persons in medical settings. Pain combined with depression has a high prevalence of 60% in clinical practice (Walker et al., 2014). Several articles confirmed that patients have been prescribed antidepressant drugs for pain relief (Walker et al., 2014; Li, 2015). The prevalence of anxiety highly depends on the considered type of pain, and the rate is between 1% and 27% for NP.

Bibliometrics is a common quantitative method for the analysis of scientific documents to determine emerging trends (Chen, 2006; Chen et al., 2012a). Numerous previous studies and reviews applied the bibliometric method to analyze trends in medical treatments, diseases, and non-pharmacological fields of research. One neuroimaging field study applied bibliometric method to analyze the trends in functional near-infrared spectroscopy in the past 20 years (Yan et al., 2020). Then, one bibliometric study analyzed the development of Alzheimer’s disease to examine China’s contribution in the field from 1988 to 2017 (Liu et al., 2019). In the non-pharmacological field, one study used the bibliometric method to analyze global publications about music therapy in the recent 20 years (Li et al., 2021). However, research on NP associated with depression or anxiety is limited. CiteSpace V is a visualization software that helps determine the development trends in a knowledge domain. Furthermore, CiteSpace software could visualize and conceptualize these research domains as science maps, identify the highly cited and core points, and detect future research trends (Chen, 2006). We utilized CiteSpace V to analyze the co-occurrence networks of authors/countries or regions/institutions, conduct an evaluation of references/keywords, and identify the top 10 most frequently cited articles from the Web of Science (WoS) database (Chen et al., 2012b).

This study aimed to present a bibliometric analysis of scientific publications on NP associated with depression or anxiety from 2000 to 2020 *via* CiteSpace V. The global scientific research about NP associated with depression or anxiety included the publication output, collaborations between institutions/countries or regions/authors, distribution of journals, subject categories of WoS, a citation burst of keywords, and the co-citation of references.

## Materials and Methods

### Data Acquisition and Search Strategy

Documents were downloaded from the WoS database on March 27, 2021. This database provides high-quality publications about various scientific areas worldwide (Durieux and Gevenois, 2010). The data retrieval strategy included the topics (anxiety or depression) AND the title (NP), and the publications between 2000 and 2020 were identified. The following search terms were employed: topics = (Anxiety OR “Anxiety Disorder*” OR “Mental health Disorder*” OR “Psychiatric Disorder*”) OR topics = (depression or depressions or depressed or despondent or gloomy or depressive) AND title = (Neuralgia$ OR Neurodynia$ OR “Neuropathic pain” OR sciatica OR “nerve crush” OR “Nerve pain$” OR “nerve cut” OR “nerve constriction” OR “nerve inflammation” OR “nerve injury” OR “nerve ligation” OR “peripheral neuropathy” OR “chronic constriction injury” OR “diabetic neuropathy”).

### Inclusion Criteria

Supplementary Figure 1 shows the inclusion criteria. Reviews and articles were selected as the document types for this study. However, letters, editorial materials, and book reviews were excluded. Furthermore, the language of publications was restricted to English. No limitation was set for species in the search. According to this advanced search, we retrieved 59 articles. Finally, 915 articles or reviews met the inclusion criteria, and these publications were investigated to obtain diverse viewpoints on the data.

### Data Extraction

Author Y-MC extracted the publications. She utilized EndNote and Microsoft Excel 2019 to perform this study. Furthermore, we extracted and labeled publication details, such as H-index, publication count, citations per paper, and citation frequency, as bibliometric indicators. H-index indicates the citation frequency of articles among these journals or researchers. Then, we concluded the top 15 subject categories for the included studies and reviews. Each review or article was labeled with at least one category.

### Statistical Methods

CiteSpace V software was employed for the analysis of articles on NP associated with depression or anxiety and the information obtained from the Thomson Reuters WoS database. CiteSpace was used for visualizing and analyzing the networks of publications as science maps (Pei et al., 2019a). The said software is utilized to analyze keywords, evolution paths, knowledge structures, and scientific development trends in the field. The visualization network map for this work contained several nodes and lines. The nodes indicate various items, such as countries or regions, institutions, authors, and cited references. The size of each node implies the number of these items. Various citation rings on the nodes indicate different years. The thickness of each citation ring represents the quality of the citation count in terms of time zones (Zheng and Wang, 2019). The lines between the nodes indicate the cooperation/co-occurrence/co-citation of publications (Chen et al., 2012a; Liang et al., 2017). In this study, CiteSpace was utilized to: (a) represent the publication growth; (b) analyze the distribution by journals and authors; (c) summarize the subject categories of WoS; (d) represent the collaborations between countries or regions and institutions; (e) perform an analysis of reference and keywords; and (f) identify the top 10 most frequently cited articles. Microsoft Excel 2019 was employed to predict the research trend on NP associated with depression or anxiety. Linear regression was applied to evaluate whether the publications decreased or increased in a period of time.

## Results

### Publication Growth and Outputs

**Figure 1:**
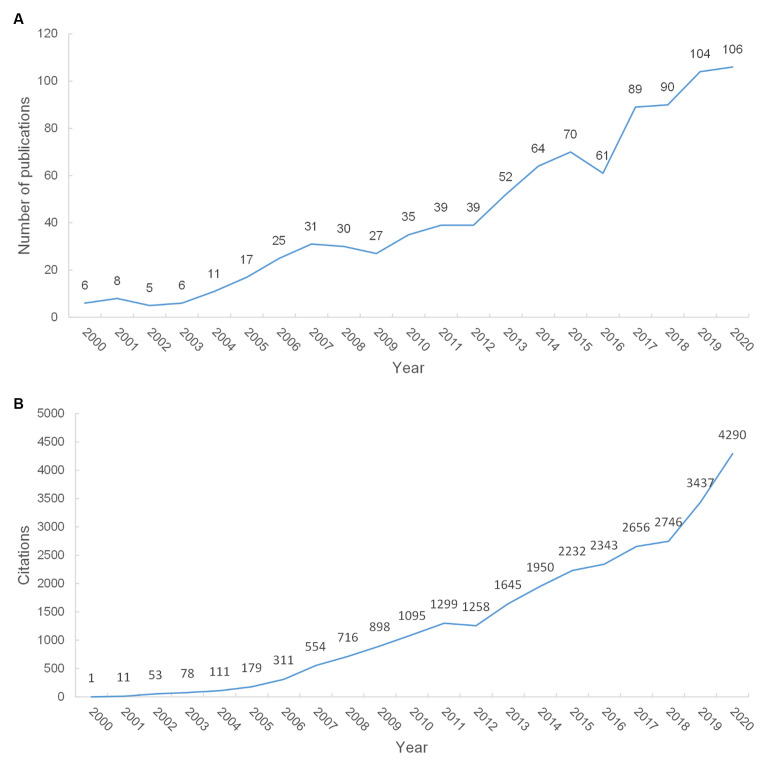
The number of publications and citations. **(A)** The number of annual publications on Neuropathic pain (NP) associated with anxiety or depression research from 2000 to 2020. **(B)** The number of annual citations on NP associated with anxiety or depression research from 2000 to 2020.

**Figure 2:**
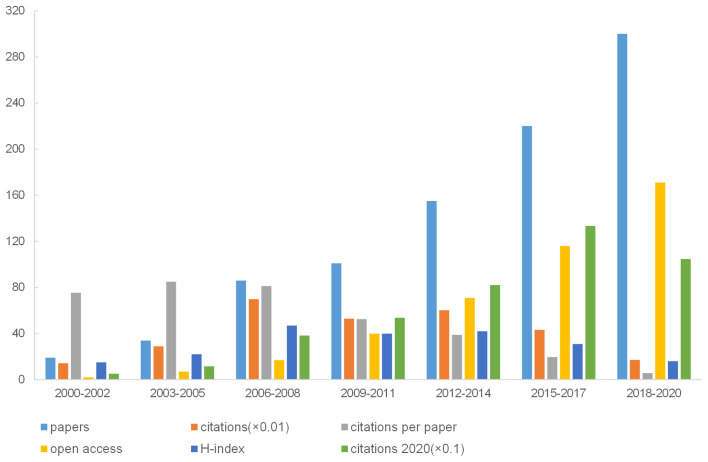
Number of articles, citations, citations per paper, open access paper, H-index, and citations in 2020 for each 3-year time period.

The remarkable increase in the number of publications in the recent 20 years implied increasing attention on NP associated with depression or anxiety. In total, 915 articles met the criteria (Supplementary Figure 1). Annual publications increased from eight in 2000 to 106 in 2020 (Figure 1A). We performed linear regression analysis and confirmed that the percentage of publications had a statistically significant increase in the study period (*p* < 0.001, *t* = 15.825). The 915 studies were cited 28,689 times (citations per paper = 31.35, H-index = 85). The number of citations also increased from 11 in 2000 to 4,290 in 2020. A statistically significant change occurred in the percentage of citations from 2000 to 2020 (*t* = 14.698, *p* < 0.001; Figure 1B). For the seven 3-year periods (2000–2002, 2003–2005, 2006–2008, 2009–2011, 2012–2014, 2015–2017, and 2018–2020), the highest number was 84.97 (from 2003 to 2005), and the largest citation number (6,992) and H-index (47) occurred in 2006–2008. In Figure 2, the largest number of citations was 1,335 (from 2015 to 2017), and the highest number of publications (300) and open access articles (171) existed in 2018–2020.

### Subject Categories of the WoS Database

**Figure 3:**
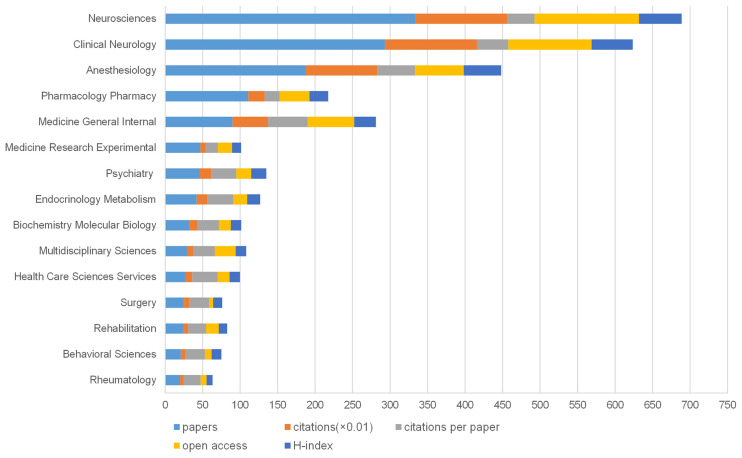
The number of articles, citations, citations per paper, open access articles, and H-index of the top 15 subject categories of Web of Science.

Each review or article was labeled with at least one subject category in the WoS database. Sixty-seven subject categories were dedicated to the 915 publications about anxiety or depression and NP. The top 15 subject categories in terms of the number of publications are shown in Figure 3. The neurosciences subject had the highest number of publications (334), followed by open access articles (139), and had the highest H-index (57). Clinical neurology had the most numerous citations (12,270). General internal medicine had the highest average citations per item (52.68). The results of linear regression analysis implied that the percentage of the top 15 subjects, namely, neurosciences, clinical neurology, anesthesiology, rehabilitation, pharmacology/pharmacy, general internal medicine, experimental medical research, psychiatry, endocrinology/metabolism, biochemistry and molecular biology, multidisciplinary sciences, healthcare science services, surgery, rehabilitation, behavioral sciences, and rheumatology, had a remarkable increase (*p* < 0.001) with time.

### Journal Distribution

In Supplementary Table 1, a total of 373 journals contributed studies on NP associated with anxiety or depression. The top 10 journals provided 25.3% (232 articles) of the publications (Table 1), and 20% of these journals belong to Q1 (top 25% of impact factor [IF] distribution). Pain (IF 2019, 5.483), the European Journal of Pain (IF 2019, 3.492), and Pain Medicine (IF 2019, 2.513) had the most numerous published studies (43 publications per journal, 4.7%). Pain had the highest IF 2019 (5.438) and H-index (34). Pain also contributed the highest number of open access articles (27) and average citations per item (71.14).

**Table 1:** The top 10 journals of origin of articles on neuropathic pain associated with anxiety or depression research.

Juornals	Papers	Citations (WoS)	Citations per paper	Open access	IF (2019)	WoS categories	Quartile	H-index
Pain	71	5,051	71.14	27	5.483	Anesthesiology; Clinical Neurology; Neurosciences	Q1;Q1;Q1	34
European Journal of Pain	32	1,099	34.34	7	3.492	Anesthesiology; Neurosciences; Clinical Neurology	Q2;Q2;Q2	20
Pain Medicine	28	1,040	37.14	24	2.513	Anesthesiology; Medicine, General and Internal	Q2;Q2	16
Neuroscience Letters	21	366	17.43	6	2.274	Neurosciences	Q3	12
Clinical Journal of Pain	17	915	53.82	2	2.893	Anesthesiology; Clinical Neurology	Q2;Q2	11
Journal of Pain Research	16	190	11.88	16	2.386	Clinical Neurology	Q3	6
Scientific Reports	13	139	10.69	13	3.998	Multidisciplinary Sciences	Q1	7
Molecular Pain	12	191	15.92	12	2.696	Neurosciences	Q3	6
Pain Practice	11	367	33.36	1	2.258	Anesthesiology; Clinical Neurology	Q3;Q3	7
Neuroscience	11	230	20.91	3	3.056	Neurosciences	Q2	8

*Abbreviations: WoS, Web of Science; IF, impact factor*.

**Figure 4:**
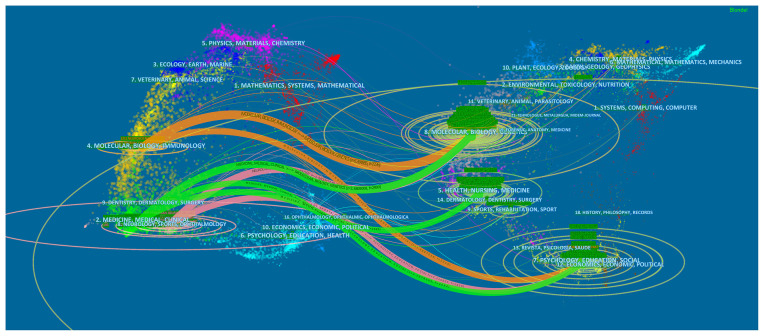
The dual-map overlay of journals related to NP associated with anxiety or depression research.

Figure 4 represents the dual map of the journals, including the map of citing and cited journals. The various lines between citing and cited journals indicate that the citation connections originated from various disciplines. The journals with the most contributions were those about molecular, biology, and immunology. The most cited journals were in molecular, biology, and genetic fields. The horizontal axes of the ellipses represent the authors, and the vertical counterparts indicate the publications.

### Distribution of Countries or Regions and Institutions

**Figure 5:**
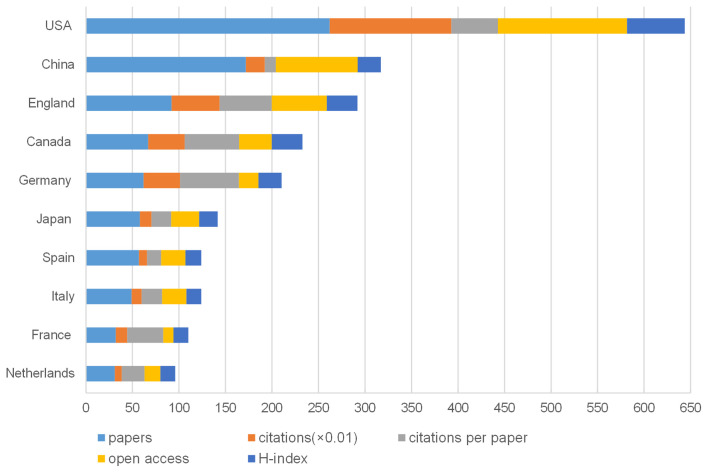
The number of articles, citations, citations per paper, open access articles, and H-index of the top 10 countries/regions.

**Figure 6:**
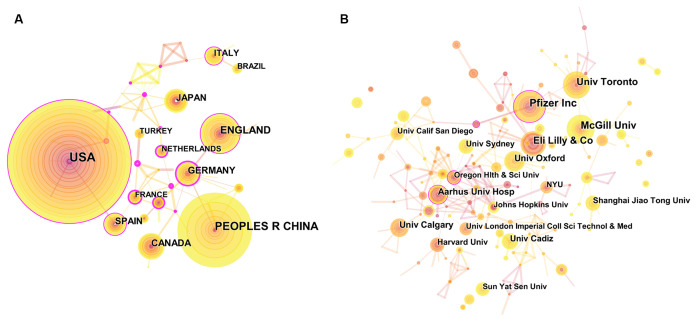
The analysis of countries/regions and institutions. **(A)** Network map of countries/regions engaged in NP associated with anxiety or depression research. **(B)** Network map of institutions engaged in NP associated with anxiety or depression research.

**Figure 7:**
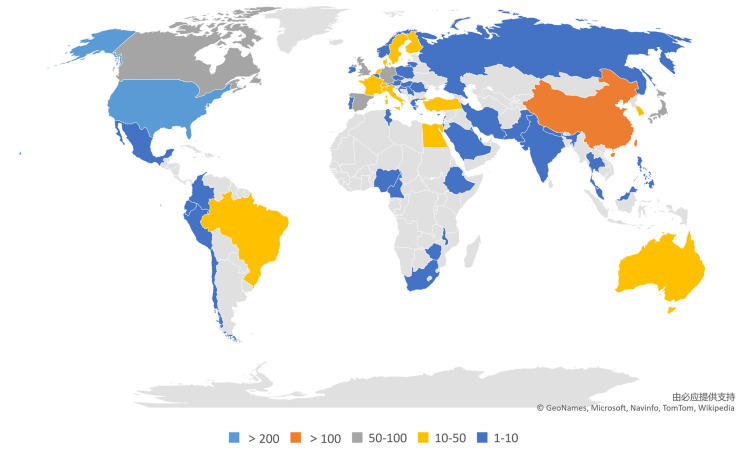
World map of total country output based on NP associated with anxiety or depression research.

Sixty-three countries or regions were involved in the 915 articles on NP associated with anxiety or depression. Figure 5 shows the number of publications along with the top 10 countries or regions. The USA had the largest number of publications (262), the largest number of citations (13,080), the most numerous open access articles (139), and the highest H-index (62). China and England followed in terms of the largest number of publications with 172 and 92 publications, respectively. Germany had the most citations per paper (63.24). Figure 6A presents the networks between countries or regions. Figure 7 presents a general view of all countries in the world map in accordance with the number of publications.

**Figure 8:**
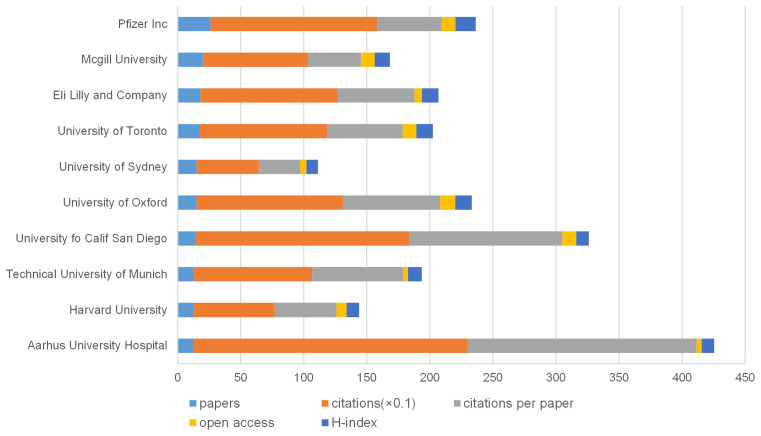
The number of articles, citations, citations per paper, open access articles, and H-index of the top 10 institutions.

In total, 1,513 institutions published studies on NP and anxiety or depression (Supplementary Table 3). The top 10 institutions are listed in Figure 8. Pfizer, Inc. had the highest number of publications (26) and H-index (16). Aarhus University Hospital had the highest number of citations (2,179) and average citations per item (181.58). Figure 6B shows the networks between institutions and indicates that cooperation was relatively rare.

### Author Distribution

**Figure 9:**
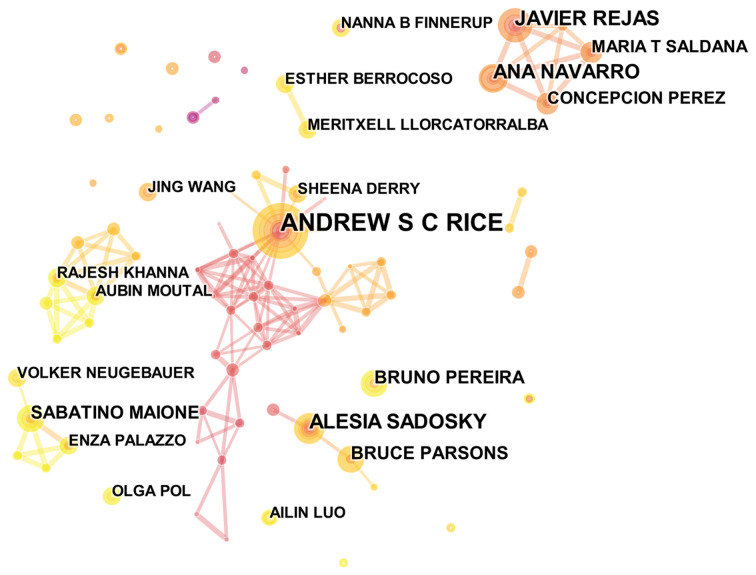
The analysis of authors. Network map of active authors contributed to NP associated with anxiety or depression research.

The 915 articles on NP with anxiety or depression were written by 4,541 authors. In terms of the number of publications, the top three most productive authors were Rice ASC (16 publications), Wang J (13 publications), and Maione S (11 publications). Rice ASC had the highest H-index (14). The connections between these productive authors are represented in Figure 9.

### Analysis of References

**Figure 10:**
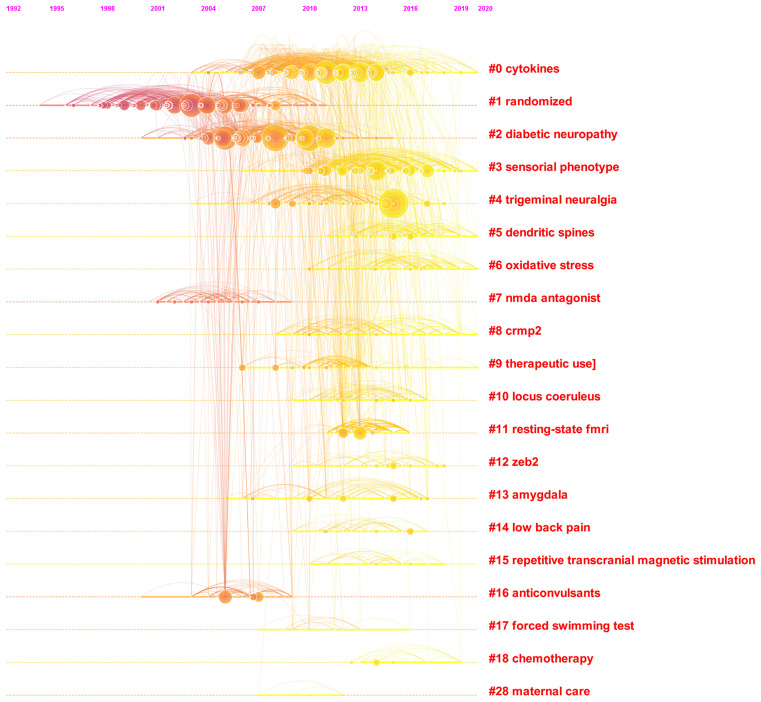
The analysis of references. Co-citation map (timeline view) of references from publications on NP associated with anxiety or depression research.

The analysis of references is an important factor in a bibliometric study. The results in Figure 10 reveal the timeline view of the co-citation analysis of references. The top 10 clusters named by the index terms in the cited references are also provided. The *Q* value implies the significance of community structure, that is, a *Q* > 0.3 corresponds to a significant community structure. The *Q* value for this work was 0.7671. “Cytokines” was the largest cluster (#0), followed by “randomized” (#1), “diabetic neuropathy” (#2), and “sensorial phenotype” (#3).

### Analysis of Keywords

**Figure 11:**
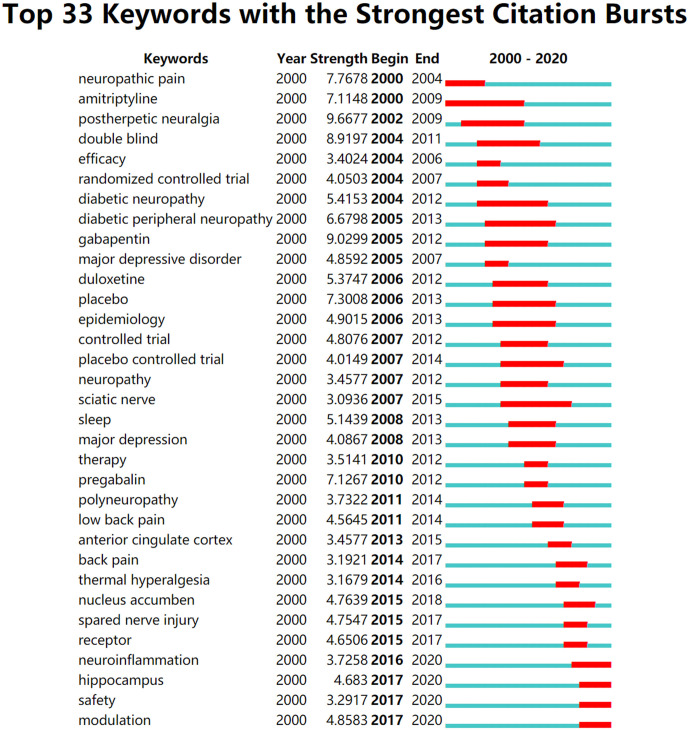
The keywords with the strongest citation bursts of publications on NP associated with anxiety or depression research.

According to the strongest citation bursts, the top 33 keywords from 2000 included NP and amitriptyline, and those from 2002 included postherpetic neuralgia (Figure 11). The keywords with the strongest citation bursts by the end of 2020 included “neuroinflammation” (2016–2020), as well as “hippocampus,” “safety,” and “modulation” (2017–2020), among the top 33 keywords (NP, amitriptyline, postherpetic neuralgia, double-blind, efficacy, randomized controlled trial, diabetic neuropathy, diabetic peripheral neuropathy, gabapentin, major depressive disorder, duloxetine, placebo, epidemiology, controlled trial, placebo-controlled trial, neuropathy, sciatic nerve, sleep, major depression, therapy, pregabalin, polyneuropathy, low back pain, anterior cingulate cortex, back pain, thermal hyperalgesia, nucleus accumben, spared nerve injury, receptor, neuroinflammation, hippocampus, safety, and modulation).

### Features of the Top 10 Most Cited Publications

The top 10 publications on NP and anxiety or depression are listed in Table 2 according to the number of citations. The citations of these publications accounted for 14.21% (4,077) of the total quantity of citations. The study “Pharmacologic management of NP: Evidence-based recommendations” by Dworkin et al. (2007) published in 2007 in Pain was the most cited one (1,251 citations). Among the top 10 articles, one (Smith et al., 2013) was published in a journal with IF ≥40 (Jama-Journal of the American Medical Association), six (Rosenstock et al., 2004; Khedr et al., 2005; Wernicke et al., 2006; Dworkin et al., 2007; Saarto and Wiffen, 2007; Yarnitsky et al., 2012) were published in journals with 5 ≤IF <10 (Cochrane Database of Systematic Reviews; Pain; Neurology; and Journal of Neurology, Neurosurgery, and Psychiatry), and three (Schmader, 2002; Gore et al., 2005; Raskin et al., 2005) were published in journals with 3 ≤ IF <5 (Clinical Journal of Pain, Pain Medicine, and Journal of Pain and Symptom Management).

**Table 2:** The top 10 articles with the most citations on neuropathic pain associated with anxiety or depression research.

Title	First author	Journal	IF (2019)	Year	Citations (WoS)	Wos Categories	Category ranking
Pharmacologic management of neuropathic pain: Evidence-based recommendations	Dworkin, RH	Pain	5.483	2007	1,251	Anesthesiology; Clinical Neurology; Neurosciences	6/32 25/204 43/272
Pregabalin for the treatment of painful diabetic peripheral neuropathy: a double-blind, placebo-controlled trial	Rosenstock, J	Pain	5.483	2004	481	Anesthesiology; Clinical Neurology; Neurosciences	6/32 25/204 43/272
Effect of Duloxetine on Pain, Function, and Quality of Life Among Patients With Chemotherapy-Induced Painful Peripheral Neuropathy A Randomized Clinical Trial	Smith, EML	Jama-Journal of the American Medical Association	45.54	2013	117	Medicine, General and Internal	3/165
Epidemiology and impact on quality of life of postherpetic neuralgia and painful diabetic neuropathy	Schmader, KE	Clinical Journal of Pain	2.893	2002	374	Anesthesiology; Clinical Neurology	13/32 89/204
A double-blind, randomized multicenter trial comparing duloxetine with placebo in the management of diabetic peripheral neuropathic pain	Raskin, J	Pain Medicine	2.513	2005	347	Anesthesiology; Medicine, General and Internal	16/32 51/165
Antidepressants for neuropathic pain	Saarto, T	Cochrane Database of Systematic Reviews	7.890	2007	337	Medicine, General and Internal	10/165
A randomized controlled trial of duloxetine in diabetic peripheral neuropathic pain	Wernicke, JF	Neurology	8.770	2006	324	Clinical Neurology	10/204
Pain severity in diabetic peripheral neuropathy is associated with patient functioning, symptom levels of anxiety and depression, and sleep	Gore, M	Journal of Pain and Symptom Management	3.077	2005	293	Clinical Neurology; Health Care Sciences and Services; Medicine, General and Internal	78/204 22/102 40/165
Conditioned pain modulation predicts duloxetine efficacy in painful diabetic neuropathy	Yarnitsky, D	Pain	5.483	2012	280	Anesthesiology; Clinical Neurology; Neurosciences	6/32 25/204 43/272
Long-lasting antalgic effects of daily sessions of repetitive transcranial magnetic stimulation in central and peripheral neuropathic pain	Khedr, EM	Journal of Neurology Neurosurgery and Psychiatry	8.263	2005	273	Clinical Neurology; Psychiatry; Surgery	12/204 8/155 3/210

## Discussion

The current study provides a bibliometric analysis of publications on NP associated with depression or anxiety from 2000 to 2020. It revealed that NP associated with depression or anxiety has been widely researched within the last two decades. Anxiety and depression occur frequently among patients with NP. Moreover, the quality of life and well-being of patients with chronic NP are more affected than those of patients with chronic non-NP, which is not caused by damaged or irritated nerves (Colloca et al., 2017). In addition, one bibliometric study provided perspectives of studies on exercise and NP (Chen and Wang, 2020). One review examined the trends of NP field studies and compared the quantity and quality of NP studies of China with other developed countries (Ye et al., 2018). The current study was the first to conduct a bibliometric analysis of the global documentary records of NP associated with depression or anxiety in the past 20 years. The results can provide suggestions to patients, scholars, educators, medical doctors, funding agencies, and policy makers.

### Global Trends of NP Associated With Depression or Anxiety

The publication–year distribution has experienced rapid growth during the past 20 years. The annual publication outputs of NP associated with depression or anxiety were categorized into four time periods. The first time period (2000–2006) had less than 30 annual publications. The second time period (2007–2015) had a steady increase in annual publications. The third time period (2016) showed a downward trend in the number of publications (61), but the output was still higher than the average in the first time period. The fourth time period was between 2017 and 2020. The year 2019 was the turning point because it was the first year when over 100 studies were published. Thus, studies on NP associated with depression or anxiety have received increasing attention in the last 20 years.

The percentage of publications increased gradually for every three-year period, and the period from 2006 to 2008 had the maximum number of citations (6,992). The 2003–2005 period had the maximum citations per paper (84.97), and 2006–2008 had the largest H-index (47) and number of publications (300). This growth in publication number does not suggest a corresponding improvement in the quality of articles because the data extraction process obtained only the number of articles in the field and could not demonstrate the quality of articles. Overall, the high values for citations only indicated that these publications received increased attention in the area.

The USA had the greatest number of publications (262), followed by China (172), England (92), Canada (67), and Germany (62). Among the top 10 countries, six were from Europe, two came from North America, and two were from Asia (Figure 5). Among the top 10 nations, nine were developed countries, and only one was a developing country. Compared with the leading countries, China may consider publishing influential articles or reviews in the field and strengthen collaboration with other countries.

A total of 1,513 institutions or universities supported the research on NP associated with depression or anxiety. In terms of the source of publications, two institutions or universities were from North America, two were from Europe, one was from England, and one was from Australia. Therefore, institutions or universities were not important relative to the cooperation of countries.

### Research Focuses on NP Combined With Depression or Anxiety

The results represented the worldwide research on NP combined with depression or anxiety from 2000 to 2020. The three main conclusions about NP associated with depression or anxiety in terms of categories (namely, the co-citation map of reference and the keywords with the strongest citation burst) were as follows. First, the top categories about NP associated with depression or anxiety include neurosciences, clinical neurology, anesthesiology, pharmacology pharmacy, medicine general internal, medicine research experimental, and psychiatry. Therefore, given the top subject categories about NP in these fields, future researchers could explore NP in the neuromodulation and neuroimmunology fields.

Second, the burst keywords in the beginning of 2000 were NP, amitriptyline, and postherpetic neuralgia. However, the burst keywords became neuroinflammation, hippocampus, safety, and modulation by the end of 2020. This change indicates that research on NP associated with depression or anxiety increasingly shifted from phenomenon to mechanism. Moreover, the top keywords with the strongest citation began in 2000 and included NP and amitriptyline. By the end of 2020, the top keywords were “postherpetic neuralgia” (2002–2009), “gabapentin” (2005–2012), and “double-blind” (2004–2011). The research frontiers can be indicated by an in-depth analysis of these keywords (Li et al., 2021).

a. Postherpetic neuralgia: Postherpetic neuralgia is a type of NP that is common among older patients after the rash of singles disappears (Pei et al., 2019b). Liu’s study compared the efficacy and safety of topical drugs for postherpetic neuralgia, and the results showed that lidocaine is the most effective drug for patients with postherpetic neuralgia (Liu et al., 2020).
b. Gabapentin: Gabapentin is one of the first-line medications for NP disorders (Wiffen et al., 2017). Many studies used oral gabapentin or gabapentin enacarbil at doses of 1800–3600 mg daily for pain relief (Wiffen et al., 2017; Moore and Gaines, 2019). Moreover, gabapentin helps release neurotransmitters and decreases neuronal excitability (Boyle et al., 2014; Chang et al., 2014).
c. Double-blind: The most effective research method is double-blind technique. This approach is particularly valuable for preventing biases due to demand characteristics or the placebo effect. A double-blind clinical trial involves human participants who are unaware of who is receiving treatment, and a placebo is provided to the control group.

Third, as shown in Table 2, the top 10 publications with the most citations are potentially meaningful in exploring research frontiers. Dworkin’s research stated that pregabalin is effective in improving pain associated with diabetic peripheral neuropathy. Pregabalin could also benefit people with mood disorders and sleep disturbance and improve their quality of life (Rosenstock et al., 2004). Schmader concluded that research involving combinations of medications and randomized controlled trials examining the treatment of central NP would become a research trend for future studies (Dworkin et al., 2007).

### Strengths and Limitations

This study was the first bibliometric analysis that included a large number of publications NP associated with depression or anxiety from the WoS database from 2000 to 2020. The 915 articles evaluated were derived from several global academic journals, and their results were considered. Aside from the distribution of countries/regions, institutions, and authors, the references, keywords, and citation bursts were also analyzed.

The publications were collected from only one specific database (WoS). Thus, some publications from other databases, such as PubMed and EBSCO, were possibly missed. However, given the essential number of publications included in this analysis, a large part of research on NP associated with depression or anxiety was most likely included. In addition, only the articles published in the English language were considered, a feature which may have induced some bias in the analysis. Furthermore, the use of clinical terms, such as depression and anxiety in the literature, is concerning. Thus, future researchers could consider conducting clinical studies to differentiate temporary sad mood and chronic clinical depression in the field. Moreover, psychiatry-related articles commonly appeared in neurology or neurosurgery journals; thus, future scientists could consider publishing articles or reviews in journals in psychiatry fields. The last limitation is that *per capita* analysis was not considered when comparing countries with different sizes and populations. This omission may produce a research bias.

## Conclusion

In this study, the bibliometric analysis provided information regarding emerging trends about publications on NP associated with depression or anxiety from 2000 to 2020. The results may be useful as a basis for helping patients with NP associated with depression or anxiety and as a foundation for further research in the field. Although this work has some limitations, it exposed the research trends on NP associated with depression or anxiety. The number of publications remarkably increased from 2000 to 2020, and the publication output increased from eight studies or reviews in 2000 to 106 studies or reviews in 2020. Additionally, future researchers can explore NP associated with depression or anxiety in the neuromodulation and neuroimmunology fields and determine the sequela and interventions of NP. Furthermore, future researchers can conclude the characteristic symptoms of NP associated with depression or anxiety. Meanwhile, future researchers could consider publishing articles or reviews on NP in psychiatry journals. Therefore, this work provides a comprehensive insight into NP associated with depression or anxiety and also provides information for researchers regarding potential collaboration with other institutions, authors, research topics, and trends.

## Author Contributions

K-L-ML and Y-MC: conceptualization and methodology. Y-MC: software, formal analysis, investigation, and data curation. K-L-ML: validation. H-YH and Y-MC: resources. K-L-ML and H-YH: writing—original draft preparation and writing—review and editing. H-YH: visualization, supervision, and project administration. H-YH and X-QW: funding acquisition. All authors contributed to the article and approved the submitted version.

## Conflict of Interest

The authors declare that the research was conducted in the absence of any commercial or financial relationships that could be construed as a potential conflict of interest.

## Publisher’s Note

All claims expressed in this article are solely those of the authors and do not necessarily represent those of their affiliated organizations, or those of the publisher, the editors and the reviewers. Any product that may be evaluated in this article, or claim that may be made by its manufacturer, is not guaranteed or endorsed by the publisher.

## Supplementary Material

SUPPLEMENTARY FIGURE 1 Overview of the paper selection process.

SUPPLEMENTARY TABLE 1 Raw data on journal sources of publications on NP associated with anxiety or depression.

SUPPLEMENTARY TABLE 2 Raw data on countries/regions involved in articles on NP associated with anxiety or depression.

SUPPLEMENTARY TABLE 3 Raw data on institutions involved in research on NP associated with anxiety or depression.
