# Supplementary figures and images for: Bibliometric Analysis of Studies on Neuropathic Pain Associated With Depression or Anxiety Published From 2000 to 2020
Source: Front Hum Neurosci. 2021 Sep 6;15:729587. doi: 10.3389/fnhum.2021.729587 (PMC8450598; doi:10.3389/fnhum.2021.729587)

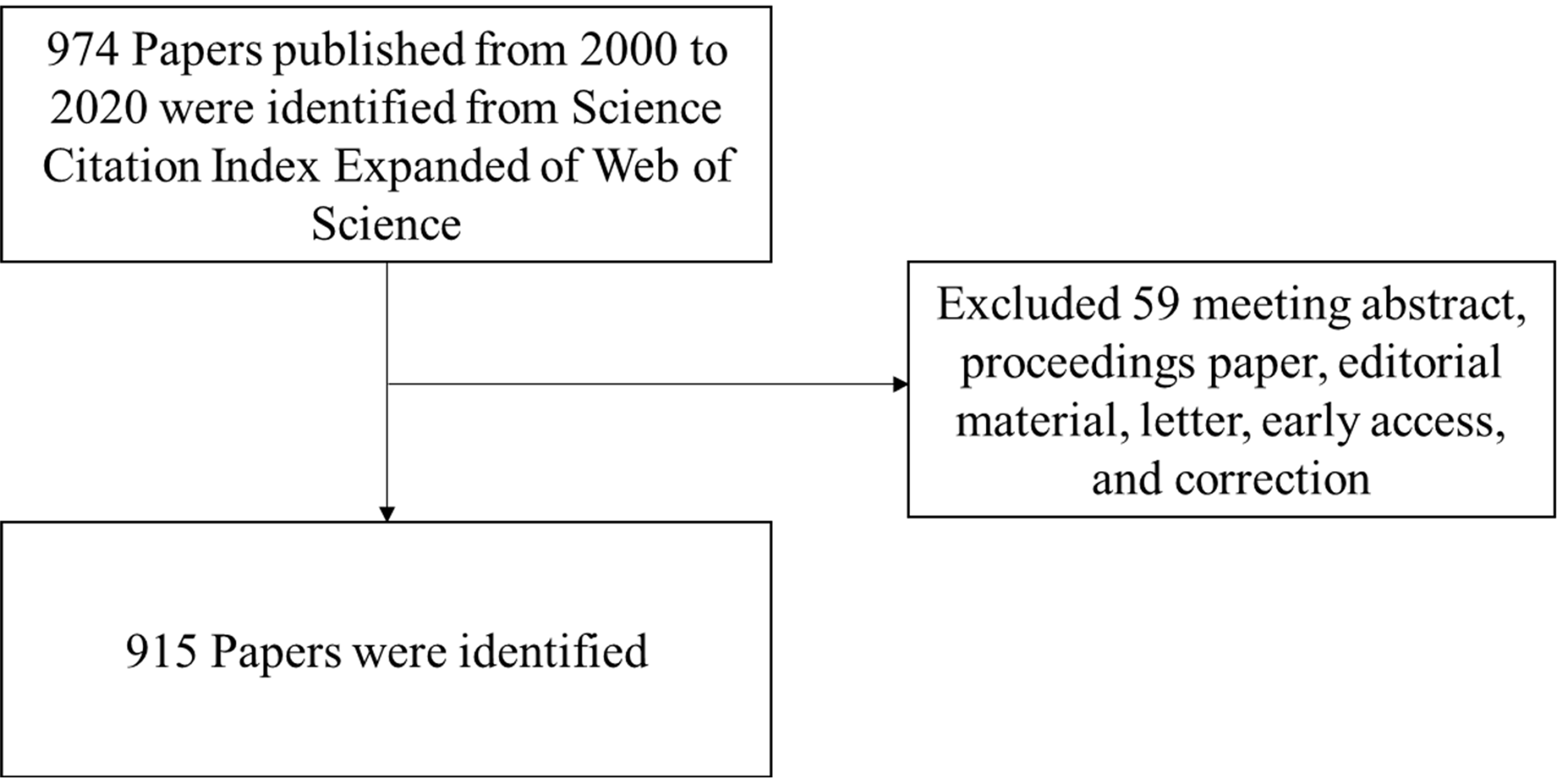

Supplement: SUPPLEMENTARY FIGURE 1 — Overview of the paper selection process. [file Image_1.TIF]
