# Supplementary material for: Bibliometric Analysis of Studies on Neuropathic Pain Associated With Depression or Anxiety Published From 2000 to 2020
Source: Front Hum Neurosci. 2021 Sep 6;15:729587. doi: 10.3389/fnhum.2021.729587 (PMC8450598; doi:10.3389/fnhum.2021.729587)
Supplement: SUPPLEMENTARY TABLE 1 — Raw data on journal sources of publications on NP associated with anxiety or depression. [file Table_1.DOCX]

**Supplementary Table 1.** Raw data on journal sources of neuropathic pain associated with anxiety or depression publications.

| **Journals** | **Records** | **% of 915** |
| --- | --- | --- |
| AIN | 71 | 7.76 |
| EUROPEAN JOURNAL OF PAIN | 32 | 3.497 |
| PAIN MEDICINE | 28 | 3.06 |
| NEUROSCIENCE LETTERS | 21 | 2.295 |
| CLINICAL JOURNAL OF PAIN | 17 | 1.858 |
| JOURNAL OF PAIN RESEARCH | 16 | 1.749 |
| SCIENTIFIC REPORTS | 13 | 1.421 |
| MOLECULAR PAIN | 12 | 1.311 |
| FRONTIERS IN PHARMACOLOGY | 11 | 1.202 |
| NEUROSCIENCE | 11 | 1.202 |
| PAIN PHYSICIAN | 11 | 1.202 |
| PAIN PRACTICE | 11 | 1.202 |
| BRAIN RESEARCH | 10 | 1.093 |
| PLOS ONE | 10 | 1.093 |
| BRAIN BEHAVIOR AND IMMUNITY | 9 | 0.984 |
| CLINICAL THERAPEUTICS | 9 | 0.984 |
| JOURNAL OF PAIN | 9 | 0.984 |
| CURRENT MEDICAL RESEARCH AND OPINION | 8 | 0.874 |
| PAIN RESEARCH MANAGEMENT | 8 | 0.874 |
| TRIALS | 8 | 0.874 |
| BMC NEUROLOGY | 7 | 0.765 |
| COCHRANE DATABASE OF SYSTEMATIC REVIEWS | 7 | 0.765 |
| JOURNAL OF NEUROSCIENCE | 7 | 0.765 |
| NEUROLOGY | 7 | 0.765 |
| NEUROPHARMACOLOGY | 7 | 0.765 |
| CLINICAL DRUG INVESTIGATION | 6 | 0.656 |
| EXPERIMENTAL NEUROLOGY | 6 | 0.656 |
| FRONTIERS IN NEUROSCIENCE | 6 | 0.656 |
| JOURNAL OF PAIN AND SYMPTOM MANAGEMENT | 6 | 0.656 |
| MEDICINE | 6 | 0.656 |
| NEUROMODULATION | 6 | 0.656 |
| INTERNATIONAL JOURNAL OF MOLECULAR SCIENCES | 5 | 0.546 |
| MEDICAL HYPOTHESES | 5 | 0.546 |
| PROGRESS IN NEURO PSYCHOPHARMACOLOGY BIOLOGICAL PSYCHIATRY | 5 | 0.546 |
| PSYCHOPHARMACOLOGY | 5 | 0.546 |
| ACTA MEDICA MEDITERRANEA | 4 | 0.437 |
| ACTA NEUROCHIRURGICA | 4 | 0.437 |
| ANESTHESIOLOGY | 4 | 0.437 |
| BEHAVIOURAL BRAIN RESEARCH | 4 | 0.437 |
| BMJ OPEN | 4 | 0.437 |
| DIABETES CARE | 4 | 0.437 |
| DIABETES RESEARCH AND CLINICAL PRACTICE | 4 | 0.437 |
| DIABETIC MEDICINE | 4 | 0.437 |
| EUROPEAN JOURNAL OF NEUROSCIENCE | 4 | 0.437 |
| EUROPEAN JOURNAL OF PHARMACOLOGY | 4 | 0.437 |
| INTERNATIONAL JOURNAL OF NEUROSCIENCE | 4 | 0.437 |
| JOURNAL OF HEADACHE AND PAIN | 4 | 0.437 |
| JOURNAL OF NEUROINFLAMMATION | 4 | 0.437 |
| JOURNAL OF THE PERIPHERAL NERVOUS SYSTEM | 4 | 0.437 |
| MAYO CLINIC PROCEEDINGS | 4 | 0.437 |
| NEUROIMAGE | 4 | 0.437 |
| PHYSIOLOGY BEHAVIOR | 4 | 0.437 |
| WORLD NEUROSURGERY | 4 | 0.437 |
| ADVANCES IN THERAPY | 3 | 0.328 |
| ANESTHESIA AND ANALGESIA | 3 | 0.328 |
| ARCHIVES OF PHYSICAL MEDICINE AND REHABILITATION | 3 | 0.328 |
| BRAIN RESEARCH BULLETIN | 3 | 0.328 |
| CLINICAL NEUROLOGY AND NEUROSURGERY | 3 | 0.328 |
| CLINICAL RHEUMATOLOGY | 3 | 0.328 |
| CURRENT DIABETES REPORTS | 3 | 0.328 |
| EVIDENCE BASED COMPLEMENTARY AND ALTERNATIVE MEDICINE | 3 | 0.328 |
| GENES BRAIN AND BEHAVIOR | 3 | 0.328 |
| IDEGGYOGYASZATI SZEMLE CLINICAL NEUROSCIENCE | 3 | 0.328 |
| JOURNAL OF CELLULAR BIOCHEMISTRY | 3 | 0.328 |
| JOURNAL OF CLINICAL MEDICINE | 3 | 0.328 |
| JOURNAL OF DIABETES AND ITS COMPLICATIONS | 3 | 0.328 |
| JOURNAL OF NEUROSCIENCE RESEARCH | 3 | 0.328 |
| LIFE SCIENCES | 3 | 0.328 |
| NEUROREPORT | 3 | 0.328 |
| NEUROSIGNALS | 3 | 0.328 |
| OSTEOARTHRITIS AND CARTILAGE | 3 | 0.328 |
| PAIN MANAGEMENT NURSING | 3 | 0.328 |
| PHARMACOLOGY BIOCHEMISTRY AND BEHAVIOR | 3 | 0.328 |
| PROCEEDINGS OF THE NATIONAL ACADEMY OF SCIENCES OF THE UNITED STATES OF AMERICA | 3 | 0.328 |
| SPINAL CORD | 3 | 0.328 |
| SUPPORTIVE CARE IN CANCER | 3 | 0.328 |
| ACTA ANAESTHESIOLOGICA SCANDINAVICA | 2 | 0.219 |
| ACTA DIABETOLOGICA | 2 | 0.219 |
| AGE AND AGEING | 2 | 0.219 |
| AMERICAN JOURNAL OF PHYSICAL MEDICINE REHABILITATION | 2 | 0.219 |
| ARCHIVES OF RHEUMATOLOGY | 2 | 0.219 |
| ARQUIVOS DE NEURO PSIQUIATRIA | 2 | 0.219 |
| BEHAVIORAL AND BRAIN FUNCTIONS | 2 | 0.219 |
| BEHAVIOURAL PHARMACOLOGY | 2 | 0.219 |
| BIOORGANIC MEDICINAL CHEMISTRY LETTERS | 2 | 0.219 |
| BMC MUSCULOSKELETAL DISORDERS | 2 | 0.219 |
| BMC NEUROSCIENCE | 2 | 0.219 |
| BRAIN STIMULATION | 2 | 0.219 |
| BRITISH JOURNAL OF ANAESTHESIA | 2 | 0.219 |
| CANADIAN MEDICAL ASSOCIATION JOURNAL | 2 | 0.219 |
| CNS DRUGS | 2 | 0.219 |
| COMPLEMENTARY THERAPIES IN MEDICINE | 2 | 0.219 |
| DIABETES | 2 | 0.219 |
| DIABETES THERAPY | 2 | 0.219 |
| DIABETES VASCULAR DISEASE RESEARCH | 2 | 0.219 |
| DRUGS AGING | 2 | 0.219 |
| EUROPEAN JOURNAL OF CANCER CARE | 2 | 0.219 |
| EUROPEAN JOURNAL OF NEUROLOGY | 2 | 0.219 |
| EUROPEAN JOURNAL OF ONCOLOGY NURSING | 2 | 0.219 |
| EUROPEAN JOURNAL OF PAIN LONDON | 2 | 0.219 |
| EUROPEAN SPINE JOURNAL | 2 | 0.219 |
| EXPERT OPINION ON PHARMACOTHERAPY | 2 | 0.219 |
| FRONTIERS IN CELLULAR NEUROSCIENCE | 2 | 0.219 |
| HEADACHE | 2 | 0.219 |
| HEALTH AND QUALITY OF LIFE OUTCOMES | 2 | 0.219 |
| INTERNATIONAL JOURNAL OF CLINICAL AND EXPERIMENTAL MEDICINE | 2 | 0.219 |
| INTERNATIONAL JOURNAL OF CLINICAL PRACTICE | 2 | 0.219 |
| INTERNATIONAL JOURNAL OF RADIATION ONCOLOGY BIOLOGY PHYSICS | 2 | 0.219 |
| INVESTIGATIVE OPHTHALMOLOGY VISUAL SCIENCE | 2 | 0.219 |
| JOINT BONE SPINE | 2 | 0.219 |
| JOURNAL OF CLINICAL NEUROLOGY | 2 | 0.219 |
| JOURNAL OF DIABETES INVESTIGATION | 2 | 0.219 |
| JOURNAL OF NEUROCHEMISTRY | 2 | 0.219 |
| JOURNAL OF NEUROLOGICAL SCIENCES TURKISH | 2 | 0.219 |
| JOURNAL OF NEUROLOGY | 2 | 0.219 |
| JOURNAL OF NEUROSURGERY | 2 | 0.219 |
| JOURNAL OF ORAL FACIAL PAIN AND HEADACHE | 2 | 0.219 |
| JOURNAL OF PSYCHOPHARMACOLOGY | 2 | 0.219 |
| JOURNAL OF PSYCHOSOMATIC RESEARCH | 2 | 0.219 |
| JOURNAL OF REHABILITATION MEDICINE | 2 | 0.219 |
| JOURNAL OF SPINAL CORD MEDICINE | 2 | 0.219 |
| MOLECULAR NEUROBIOLOGY | 2 | 0.219 |
| MUSCLE NERVE | 2 | 0.219 |
| NEUROLOGICAL SCIENCES | 2 | 0.219 |
| NEUROPSYCHIATRIC DISEASE AND TREATMENT | 2 | 0.219 |
| NEUROPSYCHOPHARMACOLOGY | 2 | 0.219 |
| NEUROSURGERY | 2 | 0.219 |
| PEPTIDES | 2 | 0.219 |
| PHARMACOLOGICAL REPORTS | 2 | 0.219 |
| PHYSIOTHERAPY CANADA | 2 | 0.219 |
| PHYTOTHERAPY RESEARCH | 2 | 0.219 |
| PM R | 2 | 0.219 |
| PRIMARY CARE DIABETES | 2 | 0.219 |
| PSYCHOLOGY HEALTH MEDICINE | 2 | 0.219 |
| QUALITY OF LIFE RESEARCH | 2 | 0.219 |
| REGIONAL ANESTHESIA AND PAIN MEDICINE | 2 | 0.219 |
| SYNAPSE | 2 | 0.219 |
| ACTA ENDOCRINOLOGICA BUCHAREST | 1 | 0.109 |
| ACTA HISTOCHEMICA | 1 | 0.109 |
| ACTA NEUROLOGICA SCANDINAVICA | 1 | 0.109 |
| ACTA NEUROPATHOLOGICA COMMUNICATIONS | 1 | 0.109 |
| ACTA RADIOLOGICA | 1 | 0.109 |
| ACTA VETERINARIA BEOGRAD | 1 | 0.109 |
| AGING CLINICAL AND EXPERIMENTAL RESEARCH | 1 | 0.109 |
| AIDS | 1 | 0.109 |
| AMERICAN JOURNAL OF HOSPICE PALLIATIVE MEDICINE | 1 | 0.109 |
| AMERICAN JOURNAL OF MANAGED CARE | 1 | 0.109 |
| AMERICAN JOURNAL OF PHYSIOLOGY RENAL PHYSIOLOGY | 1 | 0.109 |
| AMERICAN JOURNAL OF TROPICAL MEDICINE AND HYGIENE | 1 | 0.109 |
| ANADOLU PSIKIYATRI DERGISI ANATOLIAN JOURNAL OF PSYCHIATRY | 1 | 0.109 |
| ANNALS OF CLINICAL AND TRANSLATIONAL NEUROLOGY | 1 | 0.109 |
| ANNALS OF NEUROLOGY | 1 | 0.109 |
| ANNALS OF PHARMACOTHERAPY | 1 | 0.109 |
| ANNALS OF SURGERY | 1 | 0.109 |
| ANTICANCER RESEARCH | 1 | 0.109 |
| ARCHIV DER PHARMAZIE | 1 | 0.109 |
| ARTHRITIS CARE RESEARCH | 1 | 0.109 |
| AUSTRALIAN ENDODONTIC JOURNAL | 1 | 0.109 |
| AUSTRALIAN FAMILY PHYSICIAN | 1 | 0.109 |
| BANGLADESH JOURNAL OF PHARMACOLOGY | 1 | 0.109 |
| BIOCHEMICAL AND BIOPHYSICAL RESEARCH COMMUNICATIONS | 1 | 0.109 |
| BIOCHIMICA ET BIOPHYSICA ACTA BIOMEMBRANES | 1 | 0.109 |
| BIOLOGICAL PHARMACEUTICAL BULLETIN | 1 | 0.109 |
| BIOLOGICAL PSYCHIATRY | 1 | 0.109 |
| BIOMED RESEARCH INTERNATIONAL | 1 | 0.109 |
| BIOMEDICINE PHARMACOTHERAPY | 1 | 0.109 |
| BIOMETALS | 1 | 0.109 |
| BIOORGANIC MEDICINAL CHEMISTRY | 1 | 0.109 |
| BIOSCIENCE REPORTS | 1 | 0.109 |
| BMC FAMILY PRACTICE | 1 | 0.109 |
| BMC HEALTH SERVICES RESEARCH | 1 | 0.109 |
| BMC INFECTIOUS DISEASES | 1 | 0.109 |
| BMC MEDICAL RESEARCH METHODOLOGY | 1 | 0.109 |
| BMJ BRITISH MEDICAL JOURNAL | 1 | 0.109 |
| BONE JOINT JOURNAL | 1 | 0.109 |
| BRAIN | 1 | 0.109 |
| BRAZILIAN JOURNAL OF MEDICAL AND BIOLOGICAL RESEARCH | 1 | 0.109 |
| BREAST CANCER RESEARCH AND TREATMENT | 1 | 0.109 |
| BRITISH JOURNAL OF CLINICAL PHARMACOLOGY | 1 | 0.109 |
| BRITISH JOURNAL OF NEUROSURGERY | 1 | 0.109 |
| BRITISH JOURNAL OF PHARMACOLOGY | 1 | 0.109 |
| CANADIAN JOURNAL OF DIABETES | 1 | 0.109 |
| CANADIAN JOURNAL OF NEUROLOGICAL SCIENCES | 1 | 0.109 |
| CANADIAN JOURNAL OF PHYSIOLOGY AND PHARMACOLOGY | 1 | 0.109 |
| CELLS TISSUES ORGANS | 1 | 0.109 |
| CELLULAR AND MOLECULAR NEUROBIOLOGY | 1 | 0.109 |
| CEPHALALGIA | 1 | 0.109 |
| CEREBRAL CORTEX | 1 | 0.109 |
| CHINESE JOURNAL OF PHYSIOLOGY | 1 | 0.109 |
| CLINICAL INFECTIOUS DISEASES | 1 | 0.109 |
| CLINICAL NEUROPHARMACOLOGY | 1 | 0.109 |
| CLINICAL PSYCHOPHARMACOLOGY AND NEUROSCIENCE | 1 | 0.109 |
| CLINICAL TRANSLATIONAL ONCOLOGY | 1 | 0.109 |
| CNS NEUROLOGICAL DISORDERS DRUG TARGETS | 1 | 0.109 |
| CNS SPECTRUMS | 1 | 0.109 |
| CONTEMPORARY CLINICAL TRIALS | 1 | 0.109 |
| CURRENT DRUG TARGETS | 1 | 0.109 |
| CURRENT MEDICINAL CHEMISTRY | 1 | 0.109 |
| CURRENT NEUROPHARMACOLOGY | 1 | 0.109 |
| CURRENT OPINION IN INVESTIGATIONAL DRUGS | 1 | 0.109 |
| DERMATOLOGICA SINICA | 1 | 0.109 |
| DERMATOLOGY | 1 | 0.109 |
| DIABETES METABOLISM | 1 | 0.109 |
| DIABETES METABOLISM RESEARCH AND REVIEWS | 1 | 0.109 |
| DIABETES OBESITY METABOLISM | 1 | 0.109 |
| DIABETOLOGIA | 1 | 0.109 |
| DISABILITY AND REHABILITATION | 1 | 0.109 |
| DRUGS | 1 | 0.109 |
| DRUGS OF TODAY | 1 | 0.109 |
| EUROPEAN ARCHIVES OF PSYCHIATRY AND CLINICAL NEUROSCIENCE | 1 | 0.109 |
| EUROPEAN JOURNAL OF ANAESTHESIOLOGY | 1 | 0.109 |
| EUROPEAN JOURNAL OF CANCER | 1 | 0.109 |
| EUROPEAN JOURNAL OF INTEGRATIVE MEDICINE | 1 | 0.109 |
| EUROPEAN JOURNAL OF MEDICINAL CHEMISTRY | 1 | 0.109 |
| EUROPEAN NEUROPSYCHOPHARMACOLOGY | 1 | 0.109 |
| EUROPEAN REVIEW FOR MEDICAL AND PHARMACOLOGICAL SCIENCES | 1 | 0.109 |
| EXPERIMENTAL AND CLINICAL ENDOCRINOLOGY DIABETES | 1 | 0.109 |
| EXPERIMENTAL CELL RESEARCH | 1 | 0.109 |
| EXPERIMENTAL PHYSIOLOGY | 1 | 0.109 |
| EXPERT OPINION ON DRUG METABOLISM TOXICOLOGY | 1 | 0.109 |
| EXPERT OPINION ON DRUG SAFETY | 1 | 0.109 |
| EXPERT OPINION ON THERAPEUTIC TARGETS | 1 | 0.109 |
| EXPERT REVIEW OF NEUROTHERAPEUTICS | 1 | 0.109 |
| EXPERT REVIEW OF PHARMACOECONOMICS OUTCOMES RESEARCH | 1 | 0.109 |
| FRONTIERS IN BIOSCIENCE LANDMARK | 1 | 0.109 |
| FRONTIERS IN HUMAN NEUROSCIENCE | 1 | 0.109 |
| FRONTIERS IN MOLECULAR NEUROSCIENCE | 1 | 0.109 |
| FRONTIERS IN NEUROANATOMY | 1 | 0.109 |
| FRONTIERS IN NEUROLOGY | 1 | 0.109 |
| FRONTIERS IN PSYCHIATRY | 1 | 0.109 |
| FRONTIERS IN PUBLIC HEALTH | 1 | 0.109 |
| FUNCTIONAL NEUROLOGY | 1 | 0.109 |
| FUNDAMENTAL CLINICAL PHARMACOLOGY | 1 | 0.109 |
| GENE THERAPY | 1 | 0.109 |
| GERIATRIC NURSING | 1 | 0.109 |
| GERIATRICS | 1 | 0.109 |
| HEALTH EXPECTATIONS | 1 | 0.109 |
| HEALTH TECHNOLOGY ASSESSMENT | 1 | 0.109 |
| INDIAN JOURNAL OF DERMATOLOGY | 1 | 0.109 |
| INDIAN JOURNAL OF EXPERIMENTAL BIOLOGY | 1 | 0.109 |
| INTERNATIONAL JOURNAL OF ENVIRONMENTAL RESEARCH AND PUBLIC HEALTH | 1 | 0.109 |
| INTERNATIONAL JOURNAL OF MEDICAL INFORMATICS | 1 | 0.109 |
| INTERNATIONAL JOURNAL OF ORAL AND MAXILLOFACIAL SURGERY | 1 | 0.109 |
| INTERNATIONAL JOURNAL OF RHEUMATIC DISEASES | 1 | 0.109 |
| INTERNATIONAL UROLOGY AND NEPHROLOGY | 1 | 0.109 |
| JAAPA JOURNAL OF THE AMERICAN ACADEMY OF PHYSICIAN ASSISTANTS | 1 | 0.109 |
| JAMA JOURNAL OF THE AMERICAN MEDICAL ASSOCIATION | 1 | 0.109 |
| JCI INSIGHT | 1 | 0.109 |
| JCR JOURNAL OF CLINICAL RHEUMATOLOGY | 1 | 0.109 |
| JNP JOURNAL FOR NURSE PRACTITIONERS | 1 | 0.109 |
| JOURNAL OF ADVANCED NURSING | 1 | 0.109 |
| JOURNAL OF ANATOMY | 1 | 0.109 |
| JOURNAL OF ANESTHESIA | 1 | 0.109 |
| JOURNAL OF BONE AND JOINT SURGERY AMERICAN VOLUME | 1 | 0.109 |
| JOURNAL OF CELLULAR PHYSIOLOGY | 1 | 0.109 |
| JOURNAL OF CLINICAL INVESTIGATION | 1 | 0.109 |
| JOURNAL OF CLINICAL PSYCHOPHARMACOLOGY | 1 | 0.109 |
| JOURNAL OF COMPARATIVE NEUROLOGY | 1 | 0.109 |
| JOURNAL OF ECT | 1 | 0.109 |
| JOURNAL OF EVALUATION IN CLINICAL PRACTICE | 1 | 0.109 |
| JOURNAL OF GERONTOLOGICAL NURSING | 1 | 0.109 |
| JOURNAL OF INFECTION AND PUBLIC HEALTH | 1 | 0.109 |
| JOURNAL OF MEDICAL VIROLOGY | 1 | 0.109 |
| JOURNAL OF MEDICINAL CHEMISTRY | 1 | 0.109 |
| JOURNAL OF MEDICINAL FOOD | 1 | 0.109 |
| JOURNAL OF MOLECULAR NEUROSCIENCE | 1 | 0.109 |
| JOURNAL OF MUSCULOSKELETAL PAIN | 1 | 0.109 |
| JOURNAL OF NERVOUS AND MENTAL DISEASE | 1 | 0.109 |
| JOURNAL OF NEUROENDOCRINOLOGY | 1 | 0.109 |
| JOURNAL OF NEUROLOGIC PHYSICAL THERAPY | 1 | 0.109 |
| JOURNAL OF NEUROLOGY NEUROSURGERY AND PSYCHIATRY | 1 | 0.109 |
| JOURNAL OF NEUROPHYSIOLOGY | 1 | 0.109 |
| JOURNAL OF NEUROTRAUMA | 1 | 0.109 |
| JOURNAL OF NEUROVIROLOGY | 1 | 0.109 |
| JOURNAL OF NURSING RESEARCH | 1 | 0.109 |
| JOURNAL OF ORAL AND MAXILLOFACIAL SURGERY | 1 | 0.109 |
| JOURNAL OF ORAL REHABILITATION | 1 | 0.109 |
| JOURNAL OF ORAL SCIENCE | 1 | 0.109 |
| JOURNAL OF OROFACIAL PAIN | 1 | 0.109 |
| JOURNAL OF ORTHOPAEDIC SCIENCE | 1 | 0.109 |
| JOURNAL OF PALLIATIVE MEDICINE | 1 | 0.109 |
| JOURNAL OF PHARMACOLOGY AND EXPERIMENTAL THERAPEUTICS | 1 | 0.109 |
| JOURNAL OF PHARMACY AND PHARMACOLOGY | 1 | 0.109 |
| JOURNAL OF PHYSICAL THERAPY SCIENCE | 1 | 0.109 |
| JOURNAL OF PHYSIOLOGY AND PHARMACOLOGY | 1 | 0.109 |
| JOURNAL OF RECONSTRUCTIVE MICROSURGERY | 1 | 0.109 |
| JOURNAL OF RHEUMATOLOGY | 1 | 0.109 |
| JOURNAL OF SEXUAL MEDICINE | 1 | 0.109 |
| JOURNAL OF THE AMERICAN PODIATRIC MEDICAL ASSOCIATION | 1 | 0.109 |
| JOURNAL OF THE NEUROLOGICAL SCIENCES | 1 | 0.109 |
| JOURNAL OF THE PAKISTAN MEDICAL ASSOCIATION | 1 | 0.109 |
| JOURNAL OF TRACE ELEMENTS IN MEDICINE AND BIOLOGY | 1 | 0.109 |
| JOURNAL OF TRADITIONAL CHINESE MEDICINE | 1 | 0.109 |
| JOURNAL OF TRANSLATIONAL MEDICINE | 1 | 0.109 |
| KAOHSIUNG JOURNAL OF MEDICAL SCIENCES | 1 | 0.109 |
| KOREAN JOURNAL OF PAIN | 1 | 0.109 |
| LARYNGOSCOPE | 1 | 0.109 |
| LEPROSY REVIEW | 1 | 0.109 |
| MAGNETIC RESONANCE IMAGING | 1 | 0.109 |
| MARINE DRUGS | 1 | 0.109 |
| MEDECINE ET MALADIES INFECTIEUSES | 1 | 0.109 |
| MEDICAL PRINCIPLES AND PRACTICE | 1 | 0.109 |
| MEDICINA LITHUANIA | 1 | 0.109 |
| MEDICINAL CHEMISTRY | 1 | 0.109 |
| MOLECULAR BRAIN | 1 | 0.109 |
| MOLECULAR PSYCHIATRY | 1 | 0.109 |
| MOLECULES | 1 | 0.109 |
| MULTIPLE SCLEROSIS AND RELATED DISORDERS | 1 | 0.109 |
| MULTIPLE SCLEROSIS JOURNAL | 1 | 0.109 |
| NATURE COMMUNICATIONS | 1 | 0.109 |
| NATURE MEDICINE | 1 | 0.109 |
| NAUNYN SCHMIEDEBERGS ARCHIVES OF PHARMACOLOGY | 1 | 0.109 |
| NEURAL PLASTICITY | 1 | 0.109 |
| NEUROBIOLOGY OF LEARNING AND MEMORY | 1 | 0.109 |
| NEUROCHEMICAL RESEARCH | 1 | 0.109 |
| NEUROCHEMISTRY INTERNATIONAL | 1 | 0.109 |
| NEUROENDOCRINOLOGY LETTERS | 1 | 0.109 |
| NEUROEPIDEMIOLOGY | 1 | 0.109 |
| NEUROIMAGE CLINICAL | 1 | 0.109 |
| NEUROLOGIA I NEUROCHIRURGIA POLSKA | 1 | 0.109 |
| NEUROLOGIC CLINICS | 1 | 0.109 |
| NEUROLOGICAL RESEARCH | 1 | 0.109 |
| NEURON | 1 | 0.109 |
| NEUROPHYSIOLOGIE CLINIQUE CLINICAL NEUROPHYSIOLOGY | 1 | 0.109 |
| NEUROSCIENCE AND BIOBEHAVIORAL REVIEWS | 1 | 0.109 |
| NEUROSCIENCE BULLETIN | 1 | 0.109 |
| NEUROSCIENCE RESEARCH | 1 | 0.109 |
| NEUROSURGICAL FOCUS | 1 | 0.109 |
| NEUROTHERAPEUTICS | 1 | 0.109 |
| NEUROUROLOGY AND URODYNAMICS | 1 | 0.109 |
| NIGERIAN JOURNAL OF CLINICAL PRACTICE | 1 | 0.109 |
| NUTRITIONAL NEUROSCIENCE | 1 | 0.109 |
| OCCUPATIONAL AND ENVIRONMENTAL MEDICINE | 1 | 0.109 |
| ONCOLOGY | 1 | 0.109 |
| ONCOTARGET | 1 | 0.109 |
| ONCOTARGETS AND THERAPY | 1 | 0.109 |
| OPTOMETRY AND VISION SCIENCE | 1 | 0.109 |
| ORTHOPEDICS | 1 | 0.109 |
| PAIN AND THERAPY | 1 | 0.109 |
| PATIENT PATIENT CENTERED OUTCOMES RESEARCH | 1 | 0.109 |
| PATIENT PREFERENCE AND ADHERENCE | 1 | 0.109 |
| PEDIATRICS | 1 | 0.109 |
| PHARMACOLOGICAL RESEARCH | 1 | 0.109 |
| PHARMACOLOGY RESEARCH PERSPECTIVES | 1 | 0.109 |
| POPULATION HEALTH MANAGEMENT | 1 | 0.109 |
| POSTGRADUATE MEDICINE | 1 | 0.109 |
| PRIMARY CARE | 1 | 0.109 |
| PSYCHIATRIA DANUBINA | 1 | 0.109 |
| PSYCHO ONCOLOGY | 1 | 0.109 |
| PSYCHOSOMATIC MEDICINE | 1 | 0.109 |
| PSYCHOSOMATICS | 1 | 0.109 |
| PUERTO RICO HEALTH SCIENCES JOURNAL | 1 | 0.109 |
| PURINERGIC SIGNALLING | 1 | 0.109 |
| RENDICONTI LINCEI SCIENZE FISICHE E NATURALI | 1 | 0.109 |
| REVISTA BRASILEIRA DE ANESTESIOLOGIA | 1 | 0.109 |
| REVISTA DE NEUROLOGIA | 1 | 0.109 |
| RHEUMATOLOGY INTERNATIONAL | 1 | 0.109 |
| SCIENCE SIGNALING | 1 | 0.109 |
| SEMINARS IN ARTHRITIS AND RHEUMATISM | 1 | 0.109 |
| SOMATOSENSORY AND MOTOR RESEARCH | 1 | 0.109 |
| SOUTHERN MEDICAL JOURNAL | 1 | 0.109 |
| SPINE JOURNAL | 1 | 0.109 |
| SURGICAL CLINICS OF NORTH AMERICA | 1 | 0.109 |
| SYMMETRY BASEL | 1 | 0.109 |
| THERAPEUTIC DRUG MONITORING | 1 | 0.109 |
| TRANSLATIONAL PSYCHIATRY | 1 | 0.109 |
| TURKISH JOURNAL OF GERIATRICS TURK GERIATRI DERGISI | 1 | 0.109 |
| TURKISH JOURNAL OF PHYSICAL MEDICINE AND REHABILITATION | 1 | 0.109 |
| TURKIYE FIZIKSEL TIP VE REHABILITASYON DERGISI TURKISH JOURNAL OF PHYSICAL MEDICINE AND REHABILITATION | 1 | 0.109 |
| VETERINARY JOURNAL | 1 | 0.109 |
| VOJNOSANITETSKI PREGLED | 1 | 0.109 |
| WORLD JOURNAL OF SURGERY | 1 | 0.109 |
| ZEITSCHRIFT FUR RHEUMATOLOGIE | 1 | 0.109 |
