# Supplementary material for: Bibliometric Analysis of Studies on Neuropathic Pain Associated With Depression or Anxiety Published From 2000 to 2020
Source: Front Hum Neurosci. 2021 Sep 6;15:729587. doi: 10.3389/fnhum.2021.729587 (PMC8450598; doi:10.3389/fnhum.2021.729587)
Supplement: SUPPLEMENTARY TABLE 2 — Raw data on countries/regions involved in articles on NP associated with anxiety or depression. [file Table_2.DOCX]

**Supplementary Table 2**. Raw data on countries/regions involved in neuropathic pain associated with anxiety or depression research.

| **Countries/Regions** | **Records** | **% of 915** |
| --- | --- | --- |
| USA | 262 | 28.634 |
| PEOPLES R CHINA | 160 | 17.486 |
| ENGLAND | 92 | 10.055 |
| CANADA | 67 | 7.322 |
| GERMANY | 62 | 6.776 |
| JAPAN | 58 | 6.339 |
| SPAIN | 57 | 6.23 |
| ITALY | 49 | 5.355 |
| FRANCE | 32 | 3.497 |
| NETHERLANDS | 31 | 3.388 |
| TURKEY | 31 | 3.388 |
| BRAZIL | 30 | 3.279 |
| AUSTRALIA | 29 | 3.169 |
| DENMARK | 29 | 3.169 |
| SOUTH KOREA | 27 | 2.951 |
| FINLAND | 15 | 1.639 |
| TAIWAN | 13 | 1.421 |
| SWEDEN | 12 | 1.311 |
| EGYPT | 10 | 1.093 |
| SWITZERLAND | 10 | 1.093 |
| ISRAEL | 9 | 0.984 |
| GREECE | 8 | 0.874 |
| SCOTLAND | 7 | 0.765 |
| AUSTRIA | 6 | 0.656 |
| BELGIUM | 6 | 0.656 |
| IRAN | 6 | 0.656 |
| IRELAND | 6 | 0.656 |
| MEXICO | 6 | 0.656 |
| CZECH REPUBLIC | 5 | 0.546 |
| POLAND | 5 | 0.546 |
| PORTUGAL | 5 | 0.546 |
| QATAR | 5 | 0.546 |
| CROATIA | 4 | 0.437 |
| INDIA | 4 | 0.437 |
| NORWAY | 4 | 0.437 |
| RUSSIA | 4 | 0.437 |
| SERBIA | 4 | 0.437 |
| WALES | 4 | 0.437 |
| CHILE | 3 | 0.328 |
| ETHIOPIA | 3 | 0.328 |
| MALAYSIA | 3 | 0.328 |
| SINGAPORE | 3 | 0.328 |
| THAILAND | 3 | 0.328 |
| COLOMBIA | 2 | 0.219 |
| HUNGARY | 2 | 0.219 |
| PAKISTAN | 2 | 0.219 |
| PERU | 2 | 0.219 |
| PHILIPPINES | 2 | 0.219 |
| ROMANIA | 2 | 0.219 |
| SAUDI ARABIA | 2 | 0.219 |
| SOUTH AFRICA | 2 | 0.219 |
| TUNISIA | 2 | 0.219 |
| CAMEROON | 1 | 0.109 |
| CYPRUS | 1 | 0.109 |
| ECUADOR | 1 | 0.109 |
| KUWAIT | 1 | 0.109 |
| LEBANON | 1 | 0.109 |
| LITHUANIA | 1 | 0.109 |
| MALAWI | 1 | 0.109 |
| NEPAL | 1 | 0.109 |
| NIGERIA | 1 | 0.109 |
| PALESTINE | 1 | 0.109 |
| ZIMBABWE | 1 | 0.109 |
