# Supplementary material for: Bibliometric Analysis of Studies on Neuropathic Pain Associated With Depression or Anxiety Published From 2000 to 2020
Source: Front Hum Neurosci. 2021 Sep 6;15:729587. doi: 10.3389/fnhum.2021.729587 (PMC8450598; doi:10.3389/fnhum.2021.729587)
Supplement: SUPPLEMENTARY TABLE 3 — Raw data on institutions involved in research on NP associated with anxiety or depression. [file Table_3.DOCX]

**Supplementary Table 3.** Raw data on institutions involved in neuropathic pain associated with anxiety or depression research.

| **Institution** | **Records** | **% of 915** |
| --- | --- | --- |
| PFIZER INC | 26 | 2.842 |
| MCGILL UNIV | 20 | 2.186 |
| ELI LILLY CO | 18 | 1.967 |
| UNIV TORONTO | 17 | 1.858 |
| UNIV OXFORD | 15 | 1.639 |
| UNIV SYDNEY | 15 | 1.639 |
| UNIV CALIF SAN DIEGO | 14 | 1.53 |
| HARVARD UNIV | 13 | 1.421 |
| TECH UNIV MUNICH | 13 | 1.421 |
| AARHUS UNIV HOSP | 12 | 1.311 |
| NYU | 12 | 1.311 |
| UNIV CALGARY | 12 | 1.311 |
| UNIV MANCHESTER | 12 | 1.311 |
| JOHNS HOPKINS UNIV | 11 | 1.202 |
| UCL | 11 | 1.202 |
| UNIV CADIZ | 11 | 1.202 |
| UNIV HELSINKI | 11 | 1.202 |
| UNIV WASHINGTON | 11 | 1.202 |
| FOURTH MIL MED UNIV | 10 | 1.093 |
| PEKING UNIV | 10 | 1.093 |
| SHANGHAI JIAO TONG UNIV | 10 | 1.093 |
| SUN YAT SEN UNIV | 10 | 1.093 |
| UNIV CALIF SAN FRANCISCO | 10 | 1.093 |
| UNIV LONDON IMPERIAL COLL SCI TECHNOL MED | 10 | 1.093 |
| FUDAN UNIV | 9 | 0.984 |
| ICAHN SCH MED MT SINAI | 9 | 0.984 |
| NANJING MED UNIV | 9 | 0.984 |
| OREGON HLTH SCI UNIV | 9 | 0.984 |
| PFIZER ESPANA | 9 | 0.984 |
| UNIV ARIZONA | 9 | 0.984 |
| UNIV ROCHESTER | 9 | 0.984 |
| XI AN JIAO TONG UNIV | 9 | 0.984 |
| HEIDELBERG UNIV | 8 | 0.874 |
| UNIV LIVERPOOL | 8 | 0.874 |
| UNIV MIAMI | 8 | 0.874 |
| UNIV SAO PAULO | 8 | 0.874 |
| UNIV WISCONSIN | 8 | 0.874 |
| AARHUS UNIV | 7 | 0.765 |
| DUKE UNIV | 7 | 0.765 |
| EUROPEAN BIOMETR INST | 7 | 0.765 |
| IMPERIAL COLL LONDON | 7 | 0.765 |
| INSERM | 7 | 0.765 |
| KINGS COLL LONDON | 7 | 0.765 |
| MAASTRICHT UNIV | 7 | 0.765 |
| UNIV HOSP | 7 | 0.765 |
| CAPITAL MED UNIV | 6 | 0.656 |
| CHINA MED UNIV | 6 | 0.656 |
| HOSP PRINCESA | 6 | 0.656 |
| INST SALUD CARLOS III | 6 | 0.656 |
| KAROLINSKA INST | 6 | 0.656 |
| NIHON UNIV | 6 | 0.656 |
| OSAKA UNIV | 6 | 0.656 |
| PFIZER GLOBAL RES DEV | 6 | 0.656 |
| PRIMARY CARE HLTH CTR RAICES | 6 | 0.656 |
| QUEENS UNIV | 6 | 0.656 |
| UNIV NAPLES 2 | 6 | 0.656 |
| UNIV SHEFFIELD | 6 | 0.656 |
| CHINESE ACAD SCI | 5 | 0.546 |
| CHU CLERMONT FERRAND | 5 | 0.546 |
| HUAZHONG UNIV SCI TECHNOL | 5 | 0.546 |
| MAYO CLIN | 5 | 0.546 |
| MED COLL WISCONSIN | 5 | 0.546 |
| MT SINAI SCH MED | 5 | 0.546 |
| NANCHANG UNIV | 5 | 0.546 |
| SICHUAN UNIV | 5 | 0.546 |
| TEXAS TECH UNIV | 5 | 0.546 |
| UNIV AUTONOMA BARCELONA | 5 | 0.546 |
| UNIV BASQUE COUNTRY | 5 | 0.546 |
| UNIV CLERMONT AUVERGNE | 5 | 0.546 |
| UNIV COLORADO | 5 | 0.546 |
| UNIV COPENHAGEN | 5 | 0.546 |
| UNIV MICHIGAN | 5 | 0.546 |
| UNIV REY JUAN CARLOS | 5 | 0.546 |
| UNIV STRASBOURG | 5 | 0.546 |
| UPPSALA UNIV | 5 | 0.546 |
| WASHINGTON UNIV | 5 | 0.546 |
| ZHENGZHOU UNIV | 5 | 0.546 |
| AVALON HLTH SOLUT INC | 4 | 0.437 |
| BEIJING INST PHARMACOL TOXICOL | 4 | 0.437 |
| CHELSEA WESTMINSTER HOSP NHS FDN TRUST | 4 | 0.437 |
| CHIBA UNIV | 4 | 0.437 |
| HOSHI UNIV | 4 | 0.437 |
| JOHANNES GUTENBERG UNIV MAINZ | 4 | 0.437 |
| KOREA INST SCI TECHNOL | 4 | 0.437 |
| KOREA UNIV | 4 | 0.437 |
| LILLY RES LABS | 4 | 0.437 |
| NANJING UNIV | 4 | 0.437 |
| NANTONG UNIV | 4 | 0.437 |
| NIH | 4 | 0.437 |
| ODENSE UNIV HOSP | 4 | 0.437 |
| OHIO STATE UNIV | 4 | 0.437 |
| OSAKA MED COLL | 4 | 0.437 |
| PALM BEACH NEUROL CTR | 4 | 0.437 |
| PENN STATE UNIV | 4 | 0.437 |
| PFIZER GLOBAL PHARMACEUT | 4 | 0.437 |
| PFIZER JAPAN INC | 4 | 0.437 |
| REHABIL INST CHICAGO | 4 | 0.437 |
| ROYAL HALLAMSHIRE HOSP | 4 | 0.437 |
| RUSSIAN ACAD SCI | 4 | 0.437 |
| SAPIENZA UNIV ROME | 4 | 0.437 |
| SEOUL NATL UNIV | 4 | 0.437 |
| SEOUL NATL UNIV HOSP | 4 | 0.437 |
| SHEFFIELD TEACHING HOSP NHS FDN TRUST | 4 | 0.437 |
| SOUTHEAST UNIV | 4 | 0.437 |
| SUNY BUFFALO | 4 | 0.437 |
| THIRD MIL MED UNIV | 4 | 0.437 |
| UNIV CASTILLA LA MANCHA | 4 | 0.437 |
| UNIV CHINESE ACAD SCI | 4 | 0.437 |
| UNIV CRETE | 4 | 0.437 |
| UNIV GLASGOW | 4 | 0.437 |
| UNIV HLTH NETWORK | 4 | 0.437 |
| UNIV KENTUCKY | 4 | 0.437 |
| UNIV MARYLAND | 4 | 0.437 |
| UNIV NEW MEXICO | 4 | 0.437 |
| UNIV OTTAWA | 4 | 0.437 |
| UNIV TEHRAN MED SCI | 4 | 0.437 |
| UNIV TEXAS MED BRANCH | 4 | 0.437 |
| WAYNE STATE UNIV | 4 | 0.437 |
| WEILL CORNELL MED QATAR | 4 | 0.437 |
| WENZHOU MED UNIV | 4 | 0.437 |
| YONSEI UNIV | 4 | 0.437 |
| ZHEJIANG UNIV | 4 | 0.437 |
| ASSIUT UNIV HOSP | 3 | 0.328 |
| BAYLOR COLL MED | 3 | 0.328 |
| BIOMED RES INNOVAT INST CADIZ INIBICA | 3 | 0.328 |
| CATHOLIC UNIV | 3 | 0.328 |
| CENT S UNIV | 3 | 0.328 |
| CHELSEA WESTMINSTER HOSP | 3 | 0.328 |
| CHINA MED UNIV HOSP | 3 | 0.328 |
| CHINESE ACAD MED SCI | 3 | 0.328 |
| CHURCHILL HOSP | 3 | 0.328 |
| COLUMBIA UNIV | 3 | 0.328 |
| COVANCE MARKET ACCESS SERV INC | 3 | 0.328 |
| DALHOUSIE UNIV | 3 | 0.328 |
| DREXEL UNIV | 3 | 0.328 |
| EMORY UNIV | 3 | 0.328 |
| FUJIAN MED UNIV | 3 | 0.328 |
| GRUNENTHAL GMBH | 3 | 0.328 |
| HARVARD MED SCH | 3 | 0.328 |
| HOKKAIDO UNIV | 3 | 0.328 |
| HOP AMBROISE PARE | 3 | 0.328 |
| HOSP BADALONA GERMANS TRIAS PUJOL | 3 | 0.328 |
| HOSP NACL PARAPLEJ | 3 | 0.328 |
| HOSP SANTA CREU SANT PAU | 3 | 0.328 |
| HOSP UNIV PUERTA MAR | 3 | 0.328 |
| HOSP VIRGEN DE LAS NIEVES | 3 | 0.328 |
| HUBEI UNIV MED | 3 | 0.328 |
| INDIANA UNIV SCH MED | 3 | 0.328 |
| INST INVEST BIOMED ST PAU | 3 | 0.328 |
| INT CLIN RES INST | 3 | 0.328 |
| JIANGXI PROV KEY LAB AUTON NERVOUS FUNCT DIS | 3 | 0.328 |
| JINLING HOSP | 3 | 0.328 |
| JUNTENDO UNIV | 3 | 0.328 |
| KEIO UNIV | 3 | 0.328 |
| KOBE GAKUIN UNIV | 3 | 0.328 |
| KOREA UNIV SCI TECHNOL | 3 | 0.328 |
| KYUNG HEE UNIV | 3 | 0.328 |
| KYUNGPOOK NATL UNIV | 3 | 0.328 |
| LEIDEN UNIV | 3 | 0.328 |
| LILLY DEUTSCHLAND GMBH | 3 | 0.328 |
| LUDWIG MAXIMILIANS UNIV MUNCHEN | 3 | 0.328 |
| MACQUARIE UNIV | 3 | 0.328 |
| MASARYK UNIV | 3 | 0.328 |
| MEM SLOAN KETTERING CANC CTR | 3 | 0.328 |
| NATL UNIV IRELAND GALWAY | 3 | 0.328 |
| NEUROSEARCH AS | 3 | 0.328 |
| NINGBO UNIV | 3 | 0.328 |
| NORTHWESTERN UNIV | 3 | 0.328 |
| OXFORD UNIV HOSP NHS TRUST | 3 | 0.328 |
| PARIS DIDEROT UNIV | 3 | 0.328 |
| PFIZER | 3 | 0.328 |
| PFIZER LTD | 3 | 0.328 |
| POLICY ANAL INC | 3 | 0.328 |
| PRIMARY CARE HLTH CTR PUERTA ANGEL | 3 | 0.328 |
| PRIMARY CARE HLTH CTR PUERTA DEL ANGEL | 3 | 0.328 |
| RUTGERS STATE UNIV | 3 | 0.328 |
| SAPIENZA UNIV | 3 | 0.328 |
| SELCUK UNIV | 3 | 0.328 |
| SHANDONG UNIV | 3 | 0.328 |
| SHIGA UNIV MED SCI | 3 | 0.328 |
| SOOCHOW UNIV | 3 | 0.328 |
| STANFORD UNIV | 3 | 0.328 |
| STOKE MANDEVILLE HOSP | 3 | 0.328 |
| TAIPEI VET GEN HOSP | 3 | 0.328 |
| TILBURG UNIV | 3 | 0.328 |
| UCLH NHS FDN TRUST | 3 | 0.328 |
| UNIV BELGRADE | 3 | 0.328 |
| UNIV BRISTOL | 3 | 0.328 |
| UNIV BRITISH COLUMBIA | 3 | 0.328 |
| UNIV DUSSELDORF | 3 | 0.328 |
| UNIV EASTERN FINLAND | 3 | 0.328 |
| UNIV FED SANTA MARIA | 3 | 0.328 |
| UNIV HAIFA | 3 | 0.328 |
| UNIV KIEL | 3 | 0.328 |
| UNIV MALAYA | 3 | 0.328 |
| UNIV MED CTR UTRECHT | 3 | 0.328 |
| UNIV MINHO | 3 | 0.328 |
| UNIV MINNESOTA | 3 | 0.328 |
| UNIV PENN | 3 | 0.328 |
| UNIV PITTSBURGH | 3 | 0.328 |
| UNIV POMPEU FABRA | 3 | 0.328 |
| UNIV QUEENSLAND | 3 | 0.328 |
| UNIV ROMA TOR VERGATA | 3 | 0.328 |
| UNIV VERONA | 3 | 0.328 |
| UNIV VERSAILLES ST QUENTIN | 3 | 0.328 |
| UNIV VITA SALUTE SAN RAFFAELE | 3 | 0.328 |
| UNIV WESTERN ONTARIO | 3 | 0.328 |
| UNIV WURZBURG | 3 | 0.328 |
| UNIV ZURICH | 3 | 0.328 |
| VIRGINIA COMMONWEALTH UNIV | 3 | 0.328 |
| VRIJE UNIV AMSTERDAM | 3 | 0.328 |
| WALTON CTR | 3 | 0.328 |
| XUZHOU MED UNIV | 3 | 0.328 |
| AALBORG UNIV | 2 | 0.219 |
| AALBORG UNIV HOSP | 2 | 0.219 |
| ABBOTT LABS | 2 | 0.219 |
| ABT BIO PHARMA SOLUT INC | 2 | 0.219 |
| ADELANTE CTR EXPERTISE REHABIL AUDIOL | 2 | 0.219 |
| AICHI MED UNIV | 2 | 0.219 |
| ALEXANDRIA UNIV | 2 | 0.219 |
| ALLIANT INT UNIV | 2 | 0.219 |
| AMER SOC PAIN EDUCATORS | 2 | 0.219 |
| AMPHIA HOSP | 2 | 0.219 |
| AOMORI PREFECTURAL CENT HOSP | 2 | 0.219 |
| ARISTOTLE UNIV THESSALONIKI | 2 | 0.219 |
| ASIA UNIV | 2 | 0.219 |
| ASSIUT UNIV | 2 | 0.219 |
| BANGOR UNIV | 2 | 0.219 |
| BAR ILAN UNIV | 2 | 0.219 |
| BARTS LONDON QUEEN MARYS SCH MED DENT | 2 | 0.219 |
| BEHAV NEUROSCI INST INEC | 2 | 0.219 |
| BENEDICTUS KRANKENHAUS | 2 | 0.219 |
| BENEDICTUS KRANKENHAUS TUTZING | 2 | 0.219 |
| BERLIN INST HLTH | 2 | 0.219 |
| BETH ISRAEL DEACONESS MED CTR | 2 | 0.219 |
| BOEHRINGER INGELHEIM PHARMA GMBH CO KG | 2 | 0.219 |
| BRIGHAM WOMENS HOSP | 2 | 0.219 |
| CAIRO UNIV | 2 | 0.219 |
| CARDIFF UNIV | 2 | 0.219 |
| CASE WESTERN RESERVE UNIV | 2 | 0.219 |
| CASQUAR GMBH | 2 | 0.219 |
| CATHOLIC UNIV KOREA | 2 | 0.219 |
| CHARITE | 2 | 0.219 |
| CHARLES UNIV PRAGUE | 2 | 0.219 |
| CHENGDU MED COLL | 2 | 0.219 |
| CHENGDU UNIV TRADIT CHINESE MED | 2 | 0.219 |
| CHINA ACAD CHINESE MED SCI | 2 | 0.219 |
| CHINA INST SPORT SCI | 2 | 0.219 |
| CHINA JAPAN FRIENDSHIP HOSP | 2 | 0.219 |
| CHUNG ANG UNIV | 2 | 0.219 |
| CHUNGNAM NATL UNIV | 2 | 0.219 |
| CLERMONT UNIV | 2 | 0.219 |
| CLEVELAND CLIN | 2 | 0.219 |
| CNR | 2 | 0.219 |
| CNRS | 2 | 0.219 |
| COLL MED | 2 | 0.219 |
| CTR INTERDISCIPLINARY RES REHABIL SOCIAL INTEGR | 2 | 0.219 |
| DAEGU HAANY UNIV | 2 | 0.219 |
| DAIICHI SANKYO CO LTD | 2 | 0.219 |
| DANA FARBER CANC INST | 2 | 0.219 |
| DEPT PSYCHOL | 2 | 0.219 |
| EASTERN VIRGINIA MED SCH | 2 | 0.219 |
| EGE UNIV | 2 | 0.219 |
| ELI LILLY CANADA | 2 | 0.219 |
| ERU GEVHER NESIBE HOSP | 2 | 0.219 |
| FDN SANTA LUCIA | 2 | 0.219 |
| FED UNIV ABC | 2 | 0.219 |
| FIOCRUZ MS | 2 | 0.219 |
| FRENCHAY HOSP | 2 | 0.219 |
| FUKUSHIMA MED UNIV | 2 | 0.219 |
| GALILEE MED CTR | 2 | 0.219 |
| GAZI UNIV | 2 | 0.219 |
| GUANGDONG PROV KEY LAB BRAIN FUNCT DIS | 2 | 0.219 |
| GUANGZHOU UNIV CHINESE MED | 2 | 0.219 |
| GUYS ST THOMAS NHS FDN TRUST | 2 | 0.219 |
| H LUNDBECK CO AS | 2 | 0.219 |
| HAUKELAND HOSP | 2 | 0.219 |
| HIROSAKI UNIV | 2 | 0.219 |
| HLTH SERV CONSULTING CORP | 2 | 0.219 |
| HOLLAND ORTHOPAED ARTHRIT CTR | 2 | 0.219 |
| HOP LARIBOISIERE | 2 | 0.219 |
| HOSP CHENGDU UNIV TRADIT CHINESE MED | 2 | 0.219 |
| HOSP CLIN PORTO ALEGRE | 2 | 0.219 |
| HOSP UNIV LA PAZ | 2 | 0.219 |
| HOSP UNIV LA PRINCESA | 2 | 0.219 |
| ICVS 3BS PT GOVT ASSOCIATE LAB | 2 | 0.219 |
| INHA UNIV | 2 | 0.219 |
| INST CANC ESTADO SAO PAULO | 2 | 0.219 |
| INST PSYCHOL | 2 | 0.219 |
| IRCCS NEUROMED | 2 | 0.219 |
| IST SCI SAN RAFFAELE | 2 | 0.219 |
| IZMIR KATIP CELEBI UNIV | 2 | 0.219 |
| JOHN RADCLIFFE HOSP | 2 | 0.219 |
| KAISER PERMANENTE | 2 | 0.219 |
| KAROLINSKA UNIV HOSP | 2 | 0.219 |
| KATIP CELEBI UNIV | 2 | 0.219 |
| KEELE UNIV | 2 | 0.219 |
| KINKI UNIV | 2 | 0.219 |
| KUOPIO UNIV HOSP | 2 | 0.219 |
| KYOTO UNIV | 2 | 0.219 |
| LINKOPING UNIV | 2 | 0.219 |
| LIVERPOOL JOHN MOORES UNIV | 2 | 0.219 |
| LOYOLA COLL | 2 | 0.219 |
| LUNDBECK RES | 2 | 0.219 |
| LUNDBECK RES USA | 2 | 0.219 |
| MANCHESTER METROPOLITAN UNIV | 2 | 0.219 |
| MANSOURA UNIV | 2 | 0.219 |
| MASSACHUSETTS GEN HOSP | 2 | 0.219 |
| MAXIMA MED CTR | 2 | 0.219 |
| MED UNIV VIENNA | 2 | 0.219 |
| MICHAEL E DEBAKEY VA MED CTR | 2 | 0.219 |
| MUNICIPAL HOSP QINGDAO | 2 | 0.219 |
| NAGOYA CITY UNIV | 2 | 0.219 |
| NAGOYA UNIV | 2 | 0.219 |
| NATL DEF MED CTR | 2 | 0.219 |
| NATL INST HLTH CLIN EXCELLENCE | 2 | 0.219 |
| NATL INST HLTH RES WELLCOME TRUST CLIN RES FACIL | 2 | 0.219 |
| NATL TAIWAN UNIV | 2 | 0.219 |
| NATL UNIV IRELAND | 2 | 0.219 |
| NATL UNIV SINGAPORE | 2 | 0.219 |
| NATL YANG MING UNIV | 2 | 0.219 |
| NETHERLANDS CANC INST | 2 | 0.219 |
| NETHERLANDS COMPREHENS CANC ORG IKNL | 2 | 0.219 |
| NEUROSCI BEHAV INST | 2 | 0.219 |
| NEUROSCI PAIN RES INST | 2 | 0.219 |
| NOVARTIS VACCINES DIAGNOST | 2 | 0.219 |
| ONDOKUZ MAYIS UNIV | 2 | 0.219 |
| ORION CORP | 2 | 0.219 |
| OTTAWA HOSP | 2 | 0.219 |
| PAMUKKALE UNIV | 2 | 0.219 |
| PEKING UNION MED COLL | 2 | 0.219 |
| PFIZER CANADA INC | 2 | 0.219 |
| POLICLIN UNIV CATANIA | 2 | 0.219 |
| POLISH ACAD SCI | 2 | 0.219 |
| PONTIFICIA UNIV CATOLICA CHILE | 2 | 0.219 |
| QINGDAO MENTAL HLTH CTR | 2 | 0.219 |
| QINGDAO UNIV | 2 | 0.219 |
| REG HOSP VIBORG | 2 | 0.219 |
| REHABIL CTR DE HOOGSTRAAT | 2 | 0.219 |
| ROSALIND FRANKLIN UNIV MED SCI | 2 | 0.219 |
| SANGMYUNG UNIV | 2 | 0.219 |
| SAPPORO MED UNIV | 2 | 0.219 |
| SCRIPPS RES INST | 2 | 0.219 |
| SEMMELWEIS UNIV | 2 | 0.219 |
| SHANDONG FIRST MED UNIV | 2 | 0.219 |
| SHIONOGI CO LTD | 2 | 0.219 |
| SPAARNE HOSP | 2 | 0.219 |
| ST GEORGES UNIV LONDON | 2 | 0.219 |
| SUNGSHIN UNIV | 2 | 0.219 |
| SUNY COLL GENESEO | 2 | 0.219 |
| TECHNION ISRAEL INST TECHNOL | 2 | 0.219 |
| TEL AVIV UNIV | 2 | 0.219 |
| TEMPLE UNIV | 2 | 0.219 |
| TFS | 2 | 0.219 |
| TIANJIN MED UNIV | 2 | 0.219 |
| TOKYO METROPOLITAN INST MED SCI | 2 | 0.219 |
| TOKYO UNIV HOSP | 2 | 0.219 |
| TONGJI UNIV | 2 | 0.219 |
| TOYAMA UNIV | 2 | 0.219 |
| TRAKYA UNIV | 2 | 0.219 |
| TULANE UNIV | 2 | 0.219 |
| UFUK UNIV | 2 | 0.219 |
| UNESP | 2 | 0.219 |
| UNIV ADDIS ABABA | 2 | 0.219 |
| UNIV ALABAMA BIRMINGHAM | 2 | 0.219 |
| UNIV ALBERTA | 2 | 0.219 |
| UNIV BARCELONA | 2 | 0.219 |
| UNIV BERGEN | 2 | 0.219 |
| UNIV BUCKINGHAM | 2 | 0.219 |
| UNIV CAGLIARI | 2 | 0.219 |
| UNIV CALIF LOS ANGELES | 2 | 0.219 |
| UNIV CAMPANIA LUIGI VANVITELLI | 2 | 0.219 |
| UNIV CATANIA | 2 | 0.219 |
| UNIV CINCINNATI | 2 | 0.219 |
| UNIV DUNDEE | 2 | 0.219 |
| UNIV EXETER | 2 | 0.219 |
| UNIV FED PARANA | 2 | 0.219 |
| UNIV FED RIO GRANDE DO SUL | 2 | 0.219 |
| UNIV FED SANTA CATARINA | 2 | 0.219 |
| UNIV FED SAO CARLOS | 2 | 0.219 |
| UNIV FERRARA | 2 | 0.219 |
| UNIV GOTTINGEN | 2 | 0.219 |
| UNIV GUADALAJARA | 2 | 0.219 |
| UNIV HONG KONG | 2 | 0.219 |
| UNIV HOSP CLERMONT FERRAND | 2 | 0.219 |
| UNIV HOSP PUERTA MAR | 2 | 0.219 |
| UNIV HOSP SCHLESWIG HOLSTEIN | 2 | 0.219 |
| UNIV ILLINOIS | 2 | 0.219 |
| UNIV KLINIKUM SCHLESWIG HOLSTEIN | 2 | 0.219 |
| UNIV LAVAL | 2 | 0.219 |
| UNIV LEEDS | 2 | 0.219 |
| UNIV LONDON | 2 | 0.219 |
| UNIV LYON 1 | 2 | 0.219 |
| UNIV MED CTR HAMBURG EPPENDORF | 2 | 0.219 |
| UNIV MONTREAL | 2 | 0.219 |
| UNIV MUNICH | 2 | 0.219 |
| UNIV NEW SOUTH WALES | 2 | 0.219 |
| UNIV NOTTINGHAM | 2 | 0.219 |
| UNIV PARIS 05 | 2 | 0.219 |
| UNIV RIJEKA | 2 | 0.219 |
| UNIV ROMA LA SAPIENZA | 2 | 0.219 |
| UNIV S CAROLINA | 2 | 0.219 |
| UNIV SCI TECHNOL | 2 | 0.219 |
| UNIV SOUTH CHINA | 2 | 0.219 |
| UNIV TEXAS | 2 | 0.219 |
| UNIV TEXAS MD ANDERSON CANC CTR | 2 | 0.219 |
| UNIV TUBINGEN | 2 | 0.219 |
| UNIV TURIN | 2 | 0.219 |
| UNIV VALENCIA | 2 | 0.219 |
| USA ELIXIRIA BIOTECH INC | 2 | 0.219 |
| VA SAN DIEGO HEALTHCARE SYST | 2 | 0.219 |
| VANDERBILT UNIV | 2 | 0.219 |
| VRIJE UNIV BRUSSEL | 2 | 0.219 |
| WAKAYAMA MED UNIV | 2 | 0.219 |
| WALTON CTR NHS TRUST | 2 | 0.219 |
| WEST VIRGINIA UNIV | 2 | 0.219 |
| WOMENS COLL HOSP | 2 | 0.219 |
| XIAMEN UNIV | 2 | 0.219 |
| YAMAGUCHI UNIV | 2 | 0.219 |
| YANGZHOU UNIV | 2 | 0.219 |
| YESHIVA UNIV | 2 | 0.219 |
| YORK UNIV | 2 | 0.219 |
| ZHEJIANG CHINESE MED UNIV | 2 | 0.219 |
| ZUNYI MED COLL | 2 | 0.219 |
| 101ST HOSP PLA | 1 | 0.109 |
| 1ST HOSP SOCIAL SECUR SERV | 1 | 0.109 |
| 88TH HOSP PLA | 1 | 0.109 |
| AALTO UNIV | 1 | 0.109 |
| ABS ALT MOGENT | 1 | 0.109 |
| ABT BIOPHARM SOLUT INC | 1 | 0.109 |
| ABT BIOPHARMA SOLUT INC | 1 | 0.109 |
| ACAD MIL MED SCI | 1 | 0.109 |
| ADDENBROOKES HOSP | 1 | 0.109 |
| ADELANTE ZORGGRP | 1 | 0.109 |
| ADIYAMAN UNIV | 1 | 0.109 |
| ADV CLIN CONSULTAT RES CTR | 1 | 0.109 |
| ADVOCATE ILLINOIS MASONIC MED CTR | 1 | 0.109 |
| AFFILIATED HOSP | 1 | 0.109 |
| AFFILIATED HOSP MIL MED SCI | 1 | 0.109 |
| AFYON KOCATEPE UNIV | 1 | 0.109 |
| AICHI GAKUIN UNIV | 1 | 0.109 |
| AIN SHAMS UNIV | 1 | 0.109 |
| AINTREE UNIV HOSP NHS FDN TRUST | 1 | 0.109 |
| AJOU UNIV | 1 | 0.109 |
| AL AZHAR UNIV | 1 | 0.109 |
| AL QUDS UNIV | 1 | 0.109 |
| ALAN EDWARDS CTR RES PAIN | 1 | 0.109 |
| ALBERT EINSTEIN COLL MED | 1 | 0.109 |
| ALBORZ UNIV MED SCI | 1 | 0.109 |
| ALEXANDIRA UNIV | 1 | 0.109 |
| ALL AFRICA LEPROSY TB REHABIL TRAINING ALERT CT | 1 | 0.109 |
| ALLIANCE CENT OFF | 1 | 0.109 |
| ALMA MATER STUDIORUM UNIV BOLOGNA | 1 | 0.109 |
| AMBROISE PARE HOSP | 1 | 0.109 |
| AMER UNIV BEIRUT | 1 | 0.109 |
| AMERICAN SOC PAIN EDUCATORS | 1 | 0.109 |
| AMPHIA ZIEKENHUIS | 1 | 0.109 |
| ANADOLU UNIV | 1 | 0.109 |
| ANAESTHESIOL OPERAT INTENS MED ABT | 1 | 0.109 |
| ANGELINI PHARMA SPA | 1 | 0.109 |
| ANHUI CTR SURVEILLANCE BACTERIAL RESISTANCE | 1 | 0.109 |
| ANHUI MED UNIV | 1 | 0.109 |
| ANKARA NUMUNE EGITIM ARASTIRMA HASTANESI | 1 | 0.109 |
| ANKARA NUMUNE TRAINING RES HOSP | 1 | 0.109 |
| ANKARA UNIV | 1 | 0.109 |
| ANNECY REG HOSP | 1 | 0.109 |
| ANS INC | 1 | 0.109 |
| ANTALYA TRAINING RES HOSP | 1 | 0.109 |
| AOU CITT SALUTE SCI TORINO | 1 | 0.109 |
| AOU CTO | 1 | 0.109 |
| AOU S GIOVANNI BATTISTA | 1 | 0.109 |
| AOU S MARIA DELLA MISERICORDIA | 1 | 0.109 |
| ARBOR ANALYT | 1 | 0.109 |
| ARIEL UNIV | 1 | 0.109 |
| ARTHRIT RES UK PRIMARY CARE CTR | 1 | 0.109 |
| ASKLEPIOS HOSP SCHILDAUTAL | 1 | 0.109 |
| ASL SIENA | 1 | 0.109 |
| ASOCIAC MED LOS ANDES | 1 | 0.109 |
| ASSOC CIVIL IMPACTS SALUD EDUC | 1 | 0.109 |
| ASSOC IMPROVEMENT MENTAL HLTH PROGRAMMES AMH | 1 | 0.109 |
| ASTELLAS PHARMA BV | 1 | 0.109 |
| ASTELLAS PHARMA EUROPE BV | 1 | 0.109 |
| ASTELLAS PHARMA EUROPE LTD | 1 | 0.109 |
| ASTELLAS PHARMA INC | 1 | 0.109 |
| ATATURK UNIV | 1 | 0.109 |
| ATHENS NAVAL HOSP | 1 | 0.109 |
| AUSTRALIAN NATL UNIV | 1 | 0.109 |
| AUSTRALIAN NUCL SCI TECHNOL ORG | 1 | 0.109 |
| AUSTRIAN ACAD SCI | 1 | 0.109 |
| AVILES PRIMARY CARE CTR | 1 | 0.109 |
| AZIENDA OSPED UNIV | 1 | 0.109 |
| AZIENDA OSPEDALIERA UNIV SENESE | 1 | 0.109 |
| BADALONA SERV ASSISTENCIALS SA | 1 | 0.109 |
| BADALONA SERVEIS ASSISTENCIALS SA | 1 | 0.109 |
| BAHIANA SCH MED PUBL HLTH | 1 | 0.109 |
| BALL MEM HOSP | 1 | 0.109 |
| BARRY UNIV | 1 | 0.109 |
| BARTS LONDON NHS TRUST | 1 | 0.109 |
| BASKENT UNIV | 1 | 0.109 |
| BAYLOR UNIV | 1 | 0.109 |
| BAYSHORE MED CTR | 1 | 0.109 |
| BC CTR DIS CONTROL | 1 | 0.109 |
| BCN HLTH ECON OUTCOMES RES | 1 | 0.109 |
| BEGIN HOSP | 1 | 0.109 |
| BEIJING JISHUITAN HOSP | 1 | 0.109 |
| BEIJING NORMAL UNIV | 1 | 0.109 |
| BETH ISRAEL HOSP | 1 | 0.109 |
| BETHUNE INT PEACE HOSP | 1 | 0.109 |
| BETSI CADWALADR UNIV HLTH BOARD | 1 | 0.109 |
| BG UNIV HOSP | 1 | 0.109 |
| BICETRE HOSP | 1 | 0.109 |
| BICETRE UNIV HOSP | 1 | 0.109 |
| BIO5 INST | 1 | 0.109 |
| BIOGEN | 1 | 0.109 |
| BIRMINGHAM HEARTLANDS SOLIHULL NHS TRUST | 1 | 0.109 |
| BIST | 1 | 0.109 |
| BITS PILANI | 1 | 0.109 |
| BOEHRINGER INGELHEIM GMBH CO KG | 1 | 0.109 |
| BONAVISTA PHYS THERAPY | 1 | 0.109 |
| BOND UNIV | 1 | 0.109 |
| BOSTON CHILDRENS HOSP | 1 | 0.109 |
| BOSTON FDN SIGHT | 1 | 0.109 |
| BOSTON UNIV | 1 | 0.109 |
| BPKIHS | 1 | 0.109 |
| BRIGHT ROCK PATH CONSULTING LLC | 1 | 0.109 |
| BRISTOL ROYAL INFIRM GEN HOSP | 1 | 0.109 |
| BRITISH COLUMBIA INST TECHNOL | 1 | 0.109 |
| BROD GRP | 1 | 0.109 |
| BTBU | 1 | 0.109 |
| BUCKINGHAMSHIRE HEALTHCARE NHS TRUST | 1 | 0.109 |
| BUCKINGHAMSHIRE HEALTHCARE TRUST | 1 | 0.109 |
| BUNDANG CHUK HOSP | 1 | 0.109 |
| BURNABY HOSP | 1 | 0.109 |
| CAIRO UNIV HOSP | 1 | 0.109 |
| CALIF STATE UNIV | 1 | 0.109 |
| CAMPUS BIOMED UNIV ROME | 1 | 0.109 |
| CANC RES UK LIVERPOOL CANC TRIALS UNIT | 1 | 0.109 |
| CARDIOL FDN MAUGERI IRCCS | 1 | 0.109 |
| CAROL DAVILA UNIV MED PHARM | 1 | 0.109 |
| CAS KEY LAB MENTAL HLTH | 1 | 0.109 |
| CASE WESTERN RESEARVE UNIV | 1 | 0.109 |
| CATHY GEN HOSP | 1 | 0.109 |
| CEBIX AB | 1 | 0.109 |
| CENT EMEK HOSP | 1 | 0.109 |
| CENT FINLAND CENT HOSP | 1 | 0.109 |
| CENT HOSP ZHUMADIAN CITY | 1 | 0.109 |
| CENT MANCHESTER UNIV HOSP NATL HLTH SERV FDN TRUS | 1 | 0.109 |
| CENT MANCHESTER UNIV HOSP NHS FDN TRUST | 1 | 0.109 |
| CENT TAIWAN UNIV SCI TECHNOL | 1 | 0.109 |
| CENT TEXAS VET HLTH CARE SYST | 1 | 0.109 |
| CETERO RES | 1 | 0.109 |
| CHAIM SHEBA MED CTR | 1 | 0.109 |
| CHANG GUNG UNIV | 1 | 0.109 |
| CHANGWON GYEONGSANG NATL UNIV HOSP | 1 | 0.109 |
| CHANGZHOU UNIV | 1 | 0.109 |
| CHAOYANG UNIV TECHNOL | 1 | 0.109 |
| CHARITE UNIV MED BERLIN | 1 | 0.109 |
| CHENGDU FIRST PEOPLES HOSP | 1 | 0.109 |
| CHENGDU UNIV TCM | 1 | 0.109 |
| CHI MEI MED CTR | 1 | 0.109 |
| CHIA NAN UNIV PHARM SCI | 1 | 0.109 |
| CHIA YI CHRISTIAN HOSP | 1 | 0.109 |
| CHIANG MAI UNIV | 1 | 0.109 |
| CHILD ADOLESCENT PSYCHIAT DEPT | 1 | 0.109 |
| CHILDRENS HOSP | 1 | 0.109 |
| CHILDRENS HOSP BOSTON | 1 | 0.109 |
| CHILDRENS HOSP LOS ANGELES | 1 | 0.109 |
| CHINA JAPANESE FRIENDSHIP HOSP | 1 | 0.109 |
| CHINA NATL INST STANDARDIZAT | 1 | 0.109 |
| CHINESE PEOPLES AIMED POLICE FORCE | 1 | 0.109 |
| CHINESE PEOPLES LIBERAT ARMY GEN HOSP | 1 | 0.109 |
| CHONGQING MED UNIV | 1 | 0.109 |
| CHONNAM NATL UNIV | 1 | 0.109 |
| CHONNAM NATL UNIV HOSP | 1 | 0.109 |
| CHRU LILLE | 1 | 0.109 |
| CHU | 1 | 0.109 |
| CHU AMBROISE PARE | 1 | 0.109 |
| CHU BELLEVUE | 1 | 0.109 |
| CHU DUPUYTREN | 1 | 0.109 |
| CHU GRENOBLE ALPES | 1 | 0.109 |
| CHU LIMOGES | 1 | 0.109 |
| CHU PASTEUR | 1 | 0.109 |
| CHU PELLEGRIN | 1 | 0.109 |
| CHU ST ETIENNE | 1 | 0.109 |
| CINVESTAV | 1 | 0.109 |
| CINVESTAV SEDE SUR | 1 | 0.109 |
| CITY UNIV LONDON | 1 | 0.109 |
| CIUTAT SANITARIA UNIV BELLVITGE | 1 | 0.109 |
| CLIN ACAD CTR BRAGA | 1 | 0.109 |
| CLIN ANGLO AMER | 1 | 0.109 |
| CLIN CREU BLANCA | 1 | 0.109 |
| CLIN CTR KRAGUJEVAC | 1 | 0.109 |
| CLIN HOSP MERKUR | 1 | 0.109 |
| CLIN ODONTOL UNIV | 1 | 0.109 |
| CLIN PILAR ST JORDI | 1 | 0.109 |
| CLIN RES INST | 1 | 0.109 |
| CLIN ST ANDRE | 1 | 0.109 |
| CLIN STEM CELLS PTY LTD | 1 | 0.109 |
| CLINICRES | 1 | 0.109 |
| COCHIN HOSP | 1 | 0.109 |
| COLL HLTH SCI | 1 | 0.109 |
| COLUMBIA COLL | 1 | 0.109 |
| COMSATS INST INFORMAT TECHNOL | 1 | 0.109 |
| CONCORDIA UNIV | 1 | 0.109 |
| CONTINUUM CANC CTR NEW YORK BETH ISRAEL ST LUKE | 1 | 0.109 |
| CONVERGENCE PHARMACEUT LTD | 1 | 0.109 |
| COPENHAGEN UNIV HOSP | 1 | 0.109 |
| CORVINUS UNIV BUDAPEST | 1 | 0.109 |
| COVANCE HLTH ECON OUTCOMES SERV INC | 1 | 0.109 |
| CRUCES UNIV HOSP | 1 | 0.109 |
| CSIC | 1 | 0.109 |
| CTR AGING | 1 | 0.109 |
| CTR CADE | 1 | 0.109 |
| CTR COGNIT NEUROSCI | 1 | 0.109 |
| CTR DIABETOL | 1 | 0.109 |
| CTR HOSP AFFILIE UNIV QUEBEC | 1 | 0.109 |
| CTR HOSP ALPES LEMAN | 1 | 0.109 |
| CTR HOSP BEZIERS | 1 | 0.109 |
| CTR HOSP BOULOGNE SUR MER | 1 | 0.109 |
| CTR HOSP EMILE ROUX | 1 | 0.109 |
| CTR HOSP MOULINS YZEURE | 1 | 0.109 |
| CTR HOSP ST FLOUR | 1 | 0.109 |
| CTR HOSP ST JOSEPH ST LUC | 1 | 0.109 |
| CTR HOSP VICHY | 1 | 0.109 |
| CTR HOSPICE PALLAT CARE | 1 | 0.109 |
| CTR INVEST CLIN PACIFICO | 1 | 0.109 |
| CTR INVEST ESTUDIOS AVANZADOS | 1 | 0.109 |
| CTR NATL RECH SCI | 1 | 0.109 |
| CTR PAIN BRAIN | 1 | 0.109 |
| CTR PAIN TREATMENT PALLIAT CARE | 1 | 0.109 |
| CTR PAULISTA DOR | 1 | 0.109 |
| CTR RECH INST UNIV GERIATR MONTREAL | 1 | 0.109 |
| CTR SALUD ATENC PRIMARIA PUERTA ANGEL | 1 | 0.109 |
| CTR SALUD BEJAR | 1 | 0.109 |
| DA YEH UNIV | 1 | 0.109 |
| DAEGU HANNY UNIV | 1 | 0.109 |
| DAEJEON UNIV | 1 | 0.109 |
| DAIICHI SANKYO DEV LTD | 1 | 0.109 |
| DAIICHI SANKYO PHARMA DEV | 1 | 0.109 |
| DAIICHI UNIV PHARM | 1 | 0.109 |
| DALIAN MED UNIV | 1 | 0.109 |
| DALIAN UNIV | 1 | 0.109 |
| DALLAS DIABET ENDO RES CTR | 1 | 0.109 |
| DANKOOK UNIV HOSP | 1 | 0.109 |
| DAQING GRP OILFIELD GEN HOSP | 1 | 0.109 |
| DARTMOUTH COLL SCH MED | 1 | 0.109 |
| DARTMOUTH HITCHCOCK MED CTR | 1 | 0.109 |
| DARTMOUTH MED SCH | 1 | 0.109 |
| DAVAO DOCTORS HOSP | 1 | 0.109 |
| DEMOCRITUS UNIV THRACE | 1 | 0.109 |
| DEPT ANESTHESIOL | 1 | 0.109 |
| DEPT ANESTHESIOL INTENS CARE | 1 | 0.109 |
| DEPT ANESTHESIOL PERIOPERAT CARE PAIN MED | 1 | 0.109 |
| DEPT BIOCHEM MOL BIOL | 1 | 0.109 |
| DEPT CLIN NEUROSCI | 1 | 0.109 |
| DEPT NEUROL | 1 | 0.109 |
| DEPT ORTHOPED PHYS MED REHABIL | 1 | 0.109 |
| DEPT PHARMACOL | 1 | 0.109 |
| DEPT PHARMACOL TOXICOL | 1 | 0.109 |
| DERMATOL RES LAB | 1 | 0.109 |
| DERRIFORD HOSP | 1 | 0.109 |
| DIABET HEADACHE INTERVENT CTR | 1 | 0.109 |
| DIABET RES INST FUJIAN PROV | 1 | 0.109 |
| DICLE UNIV | 1 | 0.109 |
| DIV OPHTHALMOL | 1 | 0.109 |
| DOMPE FARMACEUTICI SPA | 1 | 0.109 |
| DON CARLO GNOCCHI ONLUS FDN | 1 | 0.109 |
| DR ABDURRAHMAN YURTASLAN EGITIM ARASTIRMA HASTANE | 1 | 0.109 |
| DRACENA MED SCH UNIFADRA FUNDEC | 1 | 0.109 |
| DUQUESNE UNIV | 1 | 0.109 |
| DURBAN INT CLIN RES SITE | 1 | 0.109 |
| DURHAM VA MED CTR | 1 | 0.109 |
| DURHAM VET ADM MED CTR | 1 | 0.109 |
| E CAROLINA UNIV | 1 | 0.109 |
| EAST CHESHIRE NHS TRUST | 1 | 0.109 |
| EASTMAN DENT HOSP | 1 | 0.109 |
| EBERHARD KARLS UNIV TUBINGEN | 1 | 0.109 |
| EDWARD HINES JR VET ADM MED CTR | 1 | 0.109 |
| EL AZHAR UNIV | 1 | 0.109 |
| ELI LILLY CO INC | 1 | 0.109 |
| ELI LILLY JAPAN KK | 1 | 0.109 |
| ELIASSEN GRP | 1 | 0.109 |
| ELISABETH TWEESTEDEN HOSP | 1 | 0.109 |
| ELKERLIEK HOSP | 1 | 0.109 |
| EMGO INST HLTH CARE RES | 1 | 0.109 |
| ENDO PHARMACEUT INC | 1 | 0.109 |
| ENDPOINT OUTCOMES | 1 | 0.109 |
| EOC | 1 | 0.109 |
| EPS MONASTIR | 1 | 0.109 |
| ERASMUS MC | 1 | 0.109 |
| ERASMUS SCH CTR | 1 | 0.109 |
| ERASMUS UNIV | 1 | 0.109 |
| ERNST MORITZ ARNDT UNIV GREIFSWALD | 1 | 0.109 |
| ERZURUM SITESI | 1 | 0.109 |
| ESCPI PARISTECH | 1 | 0.109 |
| ESKISEHIR OSMANGAZI UNIV | 1 | 0.109 |
| ESTEVE | 1 | 0.109 |
| ETERA MUTUAL PENS INSURANCE CO | 1 | 0.109 |
| ETSU COLL MED | 1 | 0.109 |
| EUROCLIN INST | 1 | 0.109 |
| EVAGELISMOS GEN HOSP ATHENS | 1 | 0.109 |
| EVANGELISMOS GEN HOSP | 1 | 0.109 |
| EVIDERA | 1 | 0.109 |
| EXPT ONCOL DEPT | 1 | 0.109 |
| FAC MED | 1 | 0.109 |
| FAC MED MONASTIR | 1 | 0.109 |
| FAC MED SFAX | 1 | 0.109 |
| FAIR DYNAM CONSULTING | 1 | 0.109 |
| FAIR DYNAM CONSULTING SRL | 1 | 0.109 |
| FAR EASTERN FED UNIV | 1 | 0.109 |
| FDN ADOLPHE DE ROTHSCHILD | 1 | 0.109 |
| FDN IST ITALIANO TECNOL | 1 | 0.109 |
| FDN RITA LEVI MONTALCINI | 1 | 0.109 |
| FED UNIV ALFENAS UNIFAL | 1 | 0.109 |
| FED UNIV STATE RIO DE JANEIRO | 1 | 0.109 |
| FIRST HOSP JILIN UNIV | 1 | 0.109 |
| FIZYOCARE PHYS MED CTR | 1 | 0.109 |
| FLORIDA ATLANTIC UNIV | 1 | 0.109 |
| FMUSP | 1 | 0.109 |
| FOREST RES INST | 1 | 0.109 |
| FORTH PEOPLES HOSP WUXI | 1 | 0.109 |
| FRANZ TAPPEINER HOSP | 1 | 0.109 |
| FRED HUTCHINSON CANC RES CTR | 1 | 0.109 |
| FREDERIKSBERG UNIV HOSP | 1 | 0.109 |
| FREE UNIV BERLIN | 1 | 0.109 |
| FRIEDMAN BRAIN INST | 1 | 0.109 |
| FRITZ LIPMANN INST | 1 | 0.109 |
| FU JEN CATHOLIC UNIV | 1 | 0.109 |
| FUJIAN PROV HOSP | 1 | 0.109 |
| FUKUSHIMA MED UNIV SCH MED | 1 | 0.109 |
| FUNDACAO UNIV FED GRANDE DOURADOS | 1 | 0.109 |
| FUNING PEOPLES HOSP JIANGSU | 1 | 0.109 |
| GABRIEL MONTPIED TEACHING HOSP | 1 | 0.109 |
| GACHON MED SCH | 1 | 0.109 |
| GAITONDE CTR AIDS RES EDUC | 1 | 0.109 |
| GARTNAVEL ROYAL HOSP | 1 | 0.109 |
| GAZIANTEP UNIV | 1 | 0.109 |
| GECEM | 1 | 0.109 |
| GEN HOSP AGIOS ANDREAS | 1 | 0.109 |
| GEN HOSP CHINESE PLA | 1 | 0.109 |
| GEN HOSP MESSOLONGHI | 1 | 0.109 |
| GENEPRO | 1 | 0.109 |
| GENESIS RES SERV | 1 | 0.109 |
| GENEVA UNIV HOSP | 1 | 0.109 |
| GENZYME CORP | 1 | 0.109 |
| GEORG AUGUST UNIV | 1 | 0.109 |
| GEORGE WASHINGTON UNIV | 1 | 0.109 |
| GEORGETOWN UNIV | 1 | 0.109 |
| GEORGIOS GENNIMATAS GEN HOSP ATHENS | 1 | 0.109 |
| GERMAN CANC RES CTR | 1 | 0.109 |
| GERMAN DIABET CTR | 1 | 0.109 |
| GLAXOSMITHKLINE | 1 | 0.109 |
| GLHANE MIL MED ACAD | 1 | 0.109 |
| GLOSTRUP RES INST | 1 | 0.109 |
| GOLD COAST HOSP HLTH SERV | 1 | 0.109 |
| GOTHENBURG UNIV | 1 | 0.109 |
| GRAD SCH BIOMED HLTH SCI | 1 | 0.109 |
| GRAD UNIV ADV STUDIES | 1 | 0.109 |
| GREENWICH HOSP | 1 | 0.109 |
| GRIFFITH UNIV | 1 | 0.109 |
| GRP HOSP MUTUALISTE GRENOBLE | 1 | 0.109 |
| GRUNENTHAL PHARMA SA | 1 | 0.109 |
| GUANGDONG PROV ACAD CHINESE MED SCI | 1 | 0.109 |
| GUANGDONG PROV HOSP CHINESE MED | 1 | 0.109 |
| GUANGDONG SECOND PROV GEN HOSP | 1 | 0.109 |
| GUANGXI MED UNIV | 1 | 0.109 |
| GUANGZHOU MED UNIV | 1 | 0.109 |
| GUANGZHOU MIL GEN HOSP | 1 | 0.109 |
| GUIZHOU KEY LAB ANESTHESIA ORGAN PROTECT | 1 | 0.109 |
| GULHANE MIL MED ACAD | 1 | 0.109 |
| GYEONGSANG NATL UNIV | 1 | 0.109 |
| HABIB BOURGUIBA UNIV HOSP | 1 | 0.109 |
| HACHINOHE HEIWA HOSP | 1 | 0.109 |
| HADASSAH UNIV HOSP | 1 | 0.109 |
| HALLYM UNIV | 1 | 0.109 |
| HAMAMATSU UNIV | 1 | 0.109 |
| HAMAMATSU UNIV SCH MED | 1 | 0.109 |
| HAMMEL NEUROREHABIL RES CTR | 1 | 0.109 |
| HAND CTR UTRECHT | 1 | 0.109 |
| HANGZHOU FIRST PEOPLES HOSP | 1 | 0.109 |
| HANGZHOU IVY DENT CLIN CO LTD | 1 | 0.109 |
| HANNOVER MED SCH | 1 | 0.109 |
| HANYANG UNIV | 1 | 0.109 |
| HARRAN UNIV | 1 | 0.109 |
| HARVARD CHAN SCH PUBL HLTH | 1 | 0.109 |
| HARVARD SCH PUBL HLTH | 1 | 0.109 |
| HARVARD VANGUARD MED ASSOCIATES | 1 | 0.109 |
| HCL | 1 | 0.109 |
| HEADWISE LTD | 1 | 0.109 |
| HEBREW UNIV JERUSALEM | 1 | 0.109 |
| HELSINKI UNIV HOSP | 1 | 0.109 |
| HENAN PROV HOSP TCM | 1 | 0.109 |
| HENNEPIN CTY MED CTR | 1 | 0.109 |
| HERLEV HOSP | 1 | 0.109 |
| HIROSHIMA UNIV | 1 | 0.109 |
| HLTH CTR AGIA VARVARA | 1 | 0.109 |
| HLTH CTR ANOGEIA | 1 | 0.109 |
| HLTH CTR NORTH ALGECIRAS | 1 | 0.109 |
| HLTH CTR SPILI | 1 | 0.109 |
| HLTH DIST BAHIA CADIZ LA JANDA | 1 | 0.109 |
| HLTH ECON OUTCOMES RES | 1 | 0.109 |
| HLTH OUTCOMES RES DEPT | 1 | 0.109 |
| HLTH SCI CTR | 1 | 0.109 |
| HOBA THERAPEUT | 1 | 0.109 |
| HOFFMANN LA ROCHE AG | 1 | 0.109 |
| HOFSTRA NORTHWELL HLTH | 1 | 0.109 |
| HOKKAIDO CHUO ROSAI HOSP | 1 | 0.109 |
| HOKKAIDO UNIV HOSP | 1 | 0.109 |
| HOP BELLEVUE | 1 | 0.109 |
| HOP FERNAND WIDAL | 1 | 0.109 |
| HOP HENRI MONDOR | 1 | 0.109 |
| HOP LEMAN | 1 | 0.109 |
| HOP NECKER ENFANTS MALAD | 1 | 0.109 |
| HOP NEUROL | 1 | 0.109 |
| HOP RAMON Y CAJAL | 1 | 0.109 |
| HOP ST SACREMENT | 1 | 0.109 |
| HOP UNIV | 1 | 0.109 |
| HOSP CARLOS ANDRADE MARIN | 1 | 0.109 |
| HOSP CIVILS LYON | 1 | 0.109 |
| HOSP CLIN UNIV | 1 | 0.109 |
| HOSP CLIN UNIV VIRGEN DE LA ARRIXACA | 1 | 0.109 |
| HOSP CONCOICAO | 1 | 0.109 |
| HOSP CRUCES | 1 | 0.109 |
| HOSP DEL MAR MED RES INST IMIM | 1 | 0.109 |
| HOSP DON BENITO VILLANUEVA SERENA | 1 | 0.109 |
| HOSP DONOSTIA | 1 | 0.109 |
| HOSP GEN CATALUNA | 1 | 0.109 |
| HOSP INFANTA SOFIA | 1 | 0.109 |
| HOSP LA PRINCESA | 1 | 0.109 |
| HOSP MAR | 1 | 0.109 |
| HOSP MUNICIPAL BADALONA | 1 | 0.109 |
| HOSP QUIRON MADRID | 1 | 0.109 |
| HOSP SAN RAFFAELE | 1 | 0.109 |
| HOSP SANITAS ZARZUELA | 1 | 0.109 |
| HOSP SANTA CASA DE MISERICORDIA PORTO ALEGRE | 1 | 0.109 |
| HOSP SICK CHILDREN | 1 | 0.109 |
| HOSP SIRIO LIBANES | 1 | 0.109 |
| HOSP TORRECARDENAS | 1 | 0.109 |
| HOSP TXAGORRITXU | 1 | 0.109 |
| HOSP UNIV BELLVITGE | 1 | 0.109 |
| HOSP UNIV JOSE ELEUTERIO GONZALEZ | 1 | 0.109 |
| HOSP UNIV PRINCESA | 1 | 0.109 |
| HOSP UNIV PUERTA DEL MAR | 1 | 0.109 |
| HOSP UNIV RIBERA | 1 | 0.109 |
| HOSP UNIV VIRGEN ARRIXACA | 1 | 0.109 |
| HOSP UNIV VIRGEN DE LAS NIEVES | 1 | 0.109 |
| HOSP UNIV VIRGEN ROCIO | 1 | 0.109 |
| HOSP VAIL DHEBRON | 1 | 0.109 |
| HOSP VALLE DE HEBRON | 1 | 0.109 |
| HOSP VIRGEN DE LA SALUD | 1 | 0.109 |
| HOSP VIRGEN NIEVES | 1 | 0.109 |
| HOTCHKISS BRAIN INST | 1 | 0.109 |
| HOTEL DIEU COCHIN HOSP | 1 | 0.109 |
| HUBEI UNIV NATIONALITIES | 1 | 0.109 |
| HUMBOLDT UNIV | 1 | 0.109 |
| HUNGARIAN ACAD SCI | 1 | 0.109 |
| HUNTER PAIN CLIN | 1 | 0.109 |
| HYOGO MED UNIV | 1 | 0.109 |
| HYOSAN HOSP SONGHYO HEALTHCARE FDN | 1 | 0.109 |
| ILLINOIS ONCOL RES ASSOC | 1 | 0.109 |
| IMAM KHOMEINI HOSP | 1 | 0.109 |
| IMPERIAL COLL | 1 | 0.109 |
| IMS JAPAN KK | 1 | 0.109 |
| INC RES LTD | 1 | 0.109 |
| INDIANA UNIV | 1 | 0.109 |
| INFIRMERIE PROTESTANTE LYON | 1 | 0.109 |
| INJE UNIV | 1 | 0.109 |
| INNER MONGOLIA MED UNIV | 1 | 0.109 |
| INST ANALGESIA | 1 | 0.109 |
| INST BERGONIE | 1 | 0.109 |
| INST CARDIOVASC METAB MED | 1 | 0.109 |
| INST ENDOCRINOL DIABET METAB INTERNAL MED | 1 | 0.109 |
| INST FED RIO DE JANEIRO | 1 | 0.109 |
| INST INVEST SANITARIA HOSP CLIN SAN CARLOS IDISSC | 1 | 0.109 |
| INST NACL CANCEROL | 1 | 0.109 |
| INST NEUROCIENCIAS CASTILLA LEON | 1 | 0.109 |
| INST PHARMACOL TOXICOL | 1 | 0.109 |
| INST PORTUGUES ONCOL FRANCISCO GENTIL EPE | 1 | 0.109 |
| INST RES INNOVAT BIOMED SCI PROV CADIZ | 1 | 0.109 |
| INST STAT MATH | 1 | 0.109 |
| INTER AMER FDN CLIN RES | 1 | 0.109 |
| INVENTIV HLTH | 1 | 0.109 |
| IPSWICH HOSP NHS TRUST | 1 | 0.109 |
| IRAN UNIV MED SCI | 1 | 0.109 |
| IRCCS CTR NEUROLESI BONINO PULEJO | 1 | 0.109 |
| IRCCS FDN CARLO BESTA NEUROL INST | 1 | 0.109 |
| IRCCS HUMANITAS | 1 | 0.109 |
| IRCCS IST NEUROL MEDITERRANEO NEUROMED | 1 | 0.109 |
| IST SUPER SANITA | 1 | 0.109 |
| ISTANBUL SULTAN 2 ABDULHAMID HAN TRAINING RES H | 1 | 0.109 |
| ISTINYE UNIV | 1 | 0.109 |
| IWATE MED UNIV | 1 | 0.109 |
| IZMIR DOKUZ EYLUL UNIV | 1 | 0.109 |
| IZMIR UNIV HLTH SCI | 1 | 0.109 |
| JACKSON LAB | 1 | 0.109 |
| JAGIELLONIAN UNIV | 1 | 0.109 |
| JANSSEN CILAG | 1 | 0.109 |
| JANSSEN PHARMACEUT NV | 1 | 0.109 |
| JAPAN LABOR HLTH WELF ORG | 1 | 0.109 |
| JEWISH GEN HOSP MONTREAL | 1 | 0.109 |
| JIANGSU PROV CORPS HOSP | 1 | 0.109 |
| JICHI UNIV | 1 | 0.109 |
| JINAN UNIV | 1 | 0.109 |
| JK STAT DATA CONSULTANCY | 1 | 0.109 |
| JOHN HUNTER HOSP | 1 | 0.109 |
| JOHNS HOPKINS MED INST | 1 | 0.109 |
| JOHNS HOPKINS SCH MED | 1 | 0.109 |
| JOHNS HOPKINS UNIV HOSP | 1 | 0.109 |
| JOHNSON JOHNSON PHARMACEUT RES DEV | 1 | 0.109 |
| JOHNSON JOHNSON PHARMACEUT RES DEV LLC | 1 | 0.109 |
| JOSLIN DIABET CTR | 1 | 0.109 |
| JUNTENDO TOKYO KOTO GERIATR MED CTR | 1 | 0.109 |
| KAISER FRANZ JOSEF HOSP | 1 | 0.109 |
| KAISER PERMANENTE COLORADO INST HLTH RES | 1 | 0.109 |
| KAISER PERMANENTE DIV RES | 1 | 0.109 |
| KAISER PERMANENTE MED CTR | 1 | 0.109 |
| KAISER PERMANENTE OAKLAND MED CTR | 1 | 0.109 |
| KAISER PERMANENTE SOUTH SAN FRANCISCO MED CTR | 1 | 0.109 |
| KAISERIN ELISABETH SPITAL | 1 | 0.109 |
| KAOHSIUNG MED UNIV | 1 | 0.109 |
| KAOHSIUNG MED UNIV HOSP | 1 | 0.109 |
| KAOHSIUNG VET GEN HOSP | 1 | 0.109 |
| KARAMAN DEVLET HASTANESI | 1 | 0.109 |
| KAROLINSKA UNIV | 1 | 0.109 |
| KAYSERI TRAINING RES HOSP | 1 | 0.109 |
| KEAN UNIV | 1 | 0.109 |
| KERMANSHAH UNIV MED SCI | 1 | 0.109 |
| KHOO TECK PUAT HOSP | 1 | 0.109 |
| KINDAI UNIV | 1 | 0.109 |
| KING ABDULAZIZ UNIV | 1 | 0.109 |
| KINGSTON GEN HOSP | 1 | 0.109 |
| KIO UNIV | 1 | 0.109 |
| KITASATO UNIV | 1 | 0.109 |
| KLINIKUM BOGENHAUSEN | 1 | 0.109 |
| KOCAELI DERINCE TRAINING RES HOSP | 1 | 0.109 |
| KONYANG UNIV | 1 | 0.109 |
| KOREA CANC CTR HOSP | 1 | 0.109 |
| KOREA FOOD RES INST | 1 | 0.109 |
| KOREA INST ORIENTAL MED | 1 | 0.109 |
| KUNMING MED UNIV | 1 | 0.109 |
| KUSATSU GEN HOSP | 1 | 0.109 |
| KUTAYHA KATIP CELEBI EDUC RES HOSP | 1 | 0.109 |
| KUWAIT UNIV | 1 | 0.109 |
| KYUNGPOOK NATL UNIV HOSP | 1 | 0.109 |
| KYUSHU UNIV | 1 | 0.109 |
| LA MADDALENA CANC CTR | 1 | 0.109 |
| LA TROBE UNIV | 1 | 0.109 |
| LANZHOU UNIV | 1 | 0.109 |
| LARIBOISIERE HOSP | 1 | 0.109 |
| LAWSON HLTH RES INST | 1 | 0.109 |
| LEEDS GEN INFIRM | 1 | 0.109 |
| LEIBNIZ INST NEUROBIOL | 1 | 0.109 |
| LEIGHTON HOSP | 1 | 0.109 |
| LIBRA REHABIL AUDIOL | 1 | 0.109 |
| LILLY AUSTRIA | 1 | 0.109 |
| LILLY CORP CTR | 1 | 0.109 |
| LILLY USA LLC | 1 | 0.109 |
| LINKOPING UNIV HOSP | 1 | 0.109 |
| LINYI PEOPLES HOSP | 1 | 0.109 |
| LITHUANIAN UNIV HLTH SCI | 1 | 0.109 |
| LIVERPOOL UNIV NHS HOSP TRUST | 1 | 0.109 |
| LONDON REG CANC PROGRAM | 1 | 0.109 |
| LONDON SCH HYG TROP MED | 1 | 0.109 |
| LOUIS STOKES CLEVELAND VET AFFAIRS MED CTR | 1 | 0.109 |
| LUND UNIV | 1 | 0.109 |
| LW STITT STAT SERV | 1 | 0.109 |
| MACERATA HOSP AV3 ASUR MARCHE | 1 | 0.109 |
| MACKAY MED COLL | 1 | 0.109 |
| MAIMON RES LLC | 1 | 0.109 |
| MAIN LINE HEMATOL ONCOL ASSOCIATES | 1 | 0.109 |
| MANCHESTER DIABET CTR | 1 | 0.109 |
| MANCHESTER FDN TRUST | 1 | 0.109 |
| MANCHESTER ROYAL INFIRM | 1 | 0.109 |
| MAPI | 1 | 0.109 |
| MARMARA UNIV | 1 | 0.109 |
| MATERNAL CHILD HLTH CARE HOSP ZHANGQIU | 1 | 0.109 |
| MCCAIG INST BONE JOINT HLTH | 1 | 0.109 |
| MCGILL UNIV MONTREAL | 1 | 0.109 |
| MCMASTER UNIV | 1 | 0.109 |
| MED CITY | 1 | 0.109 |
| MED CTR HAAGLANDEN | 1 | 0.109 |
| MED PK GAZIOSMANPASA HOSP | 1 | 0.109 |
| MED UNIV CTR MAASTRICHT | 1 | 0.109 |
| MED UNIV GRAZ | 1 | 0.109 |
| MED UNIV S CAROLINA | 1 | 0.109 |
| MED UNIV WARSAW | 1 | 0.109 |
| MEDVAMC 145 | 1 | 0.109 |
| MEM UNIV NEWFOUNDLAND | 1 | 0.109 |
| MERRIMACK COLL | 1 | 0.109 |
| MEXICAN SOCIAL SECUR INST IMSS | 1 | 0.109 |
| MICHIGAN STATE UNIV | 1 | 0.109 |
| MILLSAPS COLL | 1 | 0.109 |
| MINIMALLY INVAS SPINE INST | 1 | 0.109 |
| MINIST EDUC | 1 | 0.109 |
| MINIST HLTH | 1 | 0.109 |
| MIT | 1 | 0.109 |
| MMS HOLDINGS INC | 1 | 0.109 |
| MOKHURI NECK BACK HOSP | 1 | 0.109 |
| MONTREAL GEN HOSP | 1 | 0.109 |
| MT SINAI BETH ISRAEL | 1 | 0.109 |
| MT SINAI MED CTR | 1 | 0.109 |
| MUMC | 1 | 0.109 |
| MUNICH CLUSTER SYST NEUROL SYNERGY | 1 | 0.109 |
| MUSTAFA KEMAL UNIV | 1 | 0.109 |
| MVZ HUMAN GENET | 1 | 0.109 |
| MYCROFT BIOANALYT INC | 1 | 0.109 |
| N SHORE LIJ HLTH SYST | 1 | 0.109 |
| NANJING JINLING HOSP | 1 | 0.109 |
| NANJING TONGREN HOSP | 1 | 0.109 |
| NANJING UNIV CHINESE MED | 1 | 0.109 |
| NARA PREFECTURE GEN REHABIL CTR | 1 | 0.109 |
| NASTAJ SP P | 1 | 0.109 |
| NATL AIDS RES INST | 1 | 0.109 |
| NATL CANC CTR | 1 | 0.109 |
| NATL CHENG KUNG UNIV | 1 | 0.109 |
| NATL CHI NAN UNIV | 1 | 0.109 |
| NATL CHIN YI UNIV TECHNOL | 1 | 0.109 |
| NATL CHUNG CHENG UNIV | 1 | 0.109 |
| NATL CHUNG HSING UNIV | 1 | 0.109 |
| NATL HLTH SERV FDN TRUST | 1 | 0.109 |
| NATL INST ANIM SCI | 1 | 0.109 |
| NATL INST BIOMED IMAGING BIOENGN | 1 | 0.109 |
| NATL INST DENT CRANIOFACIAL RES | 1 | 0.109 |
| NATL INST ONCOL | 1 | 0.109 |
| NATL KAPODISTRIAN UNIV ATHENS | 1 | 0.109 |
| NATL RES COUNCIL ITALY | 1 | 0.109 |
| NATL TAICHUNG UNIV SCI TECHNOL | 1 | 0.109 |
| NATL TAIPEI UNIV | 1 | 0.109 |
| NATL TAIPEI UNIV NURSING HLTH SCI | 1 | 0.109 |
| NATL UNIV IRELAND UNIV COLL GALWAY | 1 | 0.109 |
| NEMA RES INC | 1 | 0.109 |
| NEURALSTEM INC | 1 | 0.109 |
| NEW MEXICO VA HEALTHCARE SYST | 1 | 0.109 |
| NEW MEXICO VA HLTH CARE SYST | 1 | 0.109 |
| NEW YORK STATE PSYCHIAT INST HOSP | 1 | 0.109 |
| NEWCASTLE UNIV | 1 | 0.109 |
| NG TENG FONG GEN HOSP | 1 | 0.109 |
| NIAID | 1 | 0.109 |
| NIEHS | 1 | 0.109 |
| NIHON BIORES INC | 1 | 0.109 |
| NIHON PHARMACEUT UNIV | 1 | 0.109 |
| NIHR WELLCOME TRUST | 1 | 0.109 |
| NIMH | 1 | 0.109 |
| NINGBO 6 HOSP | 1 | 0.109 |
| NINGXIA MED UNIV | 1 | 0.109 |
| NIPPON MED SCH | 1 | 0.109 |
| NORRIS COTTON CANC CTR | 1 | 0.109 |
| NORTH SHORE UNIV HLTH SYST | 1 | 0.109 |
| NORTHSHORE UNIV HOSP | 1 | 0.109 |
| NOWON EULJI MED CTR | 1 | 0.109 |
| OAKLAND UNIV | 1 | 0.109 |
| OBAFEMI AWOLOWO UNIV | 1 | 0.109 |
| OHIO UNIV | 1 | 0.109 |
| OKAYAMA UNIV | 1 | 0.109 |
| OKLAHOMA MED RES FDN | 1 | 0.109 |
| OLMSTED MED CTR | 1 | 0.109 |
| OLVG | 1 | 0.109 |
| OLYMPUS HLTH GRP | 1 | 0.109 |
| OPEN UNIV | 1 | 0.109 |
| OPTUM INSIGHT | 1 | 0.109 |
| ORTHOPED SPECIALISTS ALABAMA | 1 | 0.109 |
| ORTON FDN | 1 | 0.109 |
| OSAKA CTR CANC CARDIOVASC DIS PREVENT | 1 | 0.109 |
| OSAKA DENT UNIV | 1 | 0.109 |
| OSAKA OHTANI UNIV | 1 | 0.109 |
| OSAKA UNIV HOSP | 1 | 0.109 |
| OSAKA YUKIOKA COLL HLTH SCI | 1 | 0.109 |
| OSPED SS ANNUNZIATA CHIETI | 1 | 0.109 |
| OUTCOMES RES CONSULTANT | 1 | 0.109 |
| PACE COMPLET PROGRAM | 1 | 0.109 |
| PACIFIC NORTHWEST UNIV HLTH SCI | 1 | 0.109 |
| PAIN DOCTORS | 1 | 0.109 |
| PAIN MANAGEMENT INST | 1 | 0.109 |
| PAIN MANAGEMENT RES CTR PKC | 1 | 0.109 |
| PAIN REHABIL INST | 1 | 0.109 |
| PAIN RES INST | 1 | 0.109 |
| PAIN TREATMENT CTR | 1 | 0.109 |
| PANJAB UNIV | 1 | 0.109 |
| PAOLO PROCACCI FDN | 1 | 0.109 |
| PARACELSUS MED UNIV | 1 | 0.109 |
| PARIS DESCARTES UNIV | 1 | 0.109 |
| PARIS LODRON UNIV SALZBURG | 1 | 0.109 |
| PASTEUR INST IRAN | 1 | 0.109 |
| PAUL CAROLE STARK NEUROSCI RES INST | 1 | 0.109 |
| PEKING UNIV HLTH SCI CTR | 1 | 0.109 |
| PEKING UNIV PEOPLES HOSP | 1 | 0.109 |
| PENN STATE MILTON S HERSHEY MED CTR | 1 | 0.109 |
| PEOPLES HOSP NINGXIA HUI NATIONALITY AUTONOMOUS R | 1 | 0.109 |
| PEOPLES HOSP YICHUN CITY | 1 | 0.109 |
| PET SCAN CTR | 1 | 0.109 |
| PFIZER CONSULTANT | 1 | 0.109 |
| PFIZER GEP SLU | 1 | 0.109 |
| PFIZER GLOBAL OUTCOMES RES | 1 | 0.109 |
| PFIZER HELLAS | 1 | 0.109 |
| PFIZER JAPAN | 1 | 0.109 |
| PFIZER PHARMACEUT GRP | 1 | 0.109 |
| PFIZER PHARMACEUT INC | 1 | 0.109 |
| PFIZER SL | 1 | 0.109 |
| PFIZER SLU | 1 | 0.109 |
| PFIZER SPAIN | 1 | 0.109 |
| PHARMERIT BV | 1 | 0.109 |
| PHYSIOL FUNDAMENTAL NEUROSCI DEPT | 1 | 0.109 |
| PLA GEN HOSP CHENGDU MIL REG | 1 | 0.109 |
| POLICLIN A GEMELLI | 1 | 0.109 |
| POMPEU FABRA UNIV CEXS UPF | 1 | 0.109 |
| PORTUGUESE INST ONCOL PORTO | 1 | 0.109 |
| PRASAT NEUROL INST | 1 | 0.109 |
| PRESIDIO OSPED PIACENZA | 1 | 0.109 |
| PRIMARY CARE CTR LORETO PUNTALES | 1 | 0.109 |
| PRIMARY CARE CTR LORETO PUNTALES HLTH DIST BAHIA | 1 | 0.109 |
| PRIMARY CARE PRACTICE ASITES | 1 | 0.109 |
| PRIMARY CARE PRACTICE EBAROS | 1 | 0.109 |
| PRIMARY CARE PRACTICE EPISKOPI | 1 | 0.109 |
| PRIMARY CARE PRACTICE HERSONISOS | 1 | 0.109 |
| PRIMARY CARE PRACTICE MALLIA | 1 | 0.109 |
| PRIMARY CARE PRACTICE MOXOS | 1 | 0.109 |
| PRIMARY CARE PRACTICE PEZA | 1 | 0.109 |
| PRIMARY CARE PRACTICE PIRGOS | 1 | 0.109 |
| PRIMARY CARE PRACTICE SKINIA | 1 | 0.109 |
| PRIMARY CARE PRACTICE VRAXASI | 1 | 0.109 |
| PRIMARY CARE PRACTICE ZONIANA | 1 | 0.109 |
| PRINCE SATTAM BIN ABDULAZIZ UNIV | 1 | 0.109 |
| PRN CONSULTING | 1 | 0.109 |
| PROF N PAULESCU NATL INST DIABET NUTR METAB DIS | 1 | 0.109 |
| PROHEALTH CARE ASSOCIATES | 1 | 0.109 |
| PUERTA DEL ANGEL PRIMARY CARE HLTH CTR | 1 | 0.109 |
| PUERTA DEL MAR UNIV HOSP | 1 | 0.109 |
| PUSAN NATL UNIV | 1 | 0.109 |
| QATAR FDN | 1 | 0.109 |
| QD CONSULTING LLC | 1 | 0.109 |
| QINGHAI UNIV | 1 | 0.109 |
| QUEEN ELIZABETH COLL MED JOHNS HOPKINS PROJECT | 1 | 0.109 |
| QUEEN MARY UNIV LONDON | 1 | 0.109 |
| QUEENSLAND UNIV TECHNOL | 1 | 0.109 |
| RADBOUD UNIV NIJMEGEN | 1 | 0.109 |
| RAMBAM HLTH CARE CAMPUS | 1 | 0.109 |
| RAMBAM MED CTR | 1 | 0.109 |
| RANGSIT UNIV | 1 | 0.109 |
| RAZI UNIV HOSP | 1 | 0.109 |
| RED CROSS ATHENS HOSP | 1 | 0.109 |
| RED CROSS HOSP | 1 | 0.109 |
| REG HOSP PARDUBICE | 1 | 0.109 |
| REGULONIX LLC | 1 | 0.109 |
| REHABIL CTR ROESSINGH | 1 | 0.109 |
| REHABIL INST MICHIGAN | 1 | 0.109 |
| RICK HANSEN INST | 1 | 0.109 |
| RIJNDAM REHABIL CTR | 1 | 0.109 |
| RMIT UNIV | 1 | 0.109 |
| ROSOMOFF PAIN CTR | 1 | 0.109 |
| ROUEN UNIV HOSP | 1 | 0.109 |
| ROYAL DEVON EXETER HOSP | 1 | 0.109 |
| ROYAL LIVERPOOL BROADGREEN UNIV NHS HOSP TRUST | 1 | 0.109 |
| ROYAL MELBOURNE HOSP | 1 | 0.109 |
| RUHR UNIV | 1 | 0.109 |
| RUHR UNIV BOCHUM | 1 | 0.109 |
| RUMMO HOSP | 1 | 0.109 |
| RUNMC | 1 | 0.109 |
| RUSH UNIV | 1 | 0.109 |
| RUTGERS SCH DENT MED | 1 | 0.109 |
| RYAN HEADACHE CTR | 1 | 0.109 |
| SADA HOSP | 1 | 0.109 |
| SAHLGRENS UNIV HOSP | 1 | 0.109 |
| SAHMYOOK UNIV | 1 | 0.109 |
| SAKAI CITY MED CTR | 1 | 0.109 |
| SALK INST BIOL SCI | 1 | 0.109 |
| SAMITHIVET SUKHUMVIT HOSP | 1 | 0.109 |
| SAMSUN EGITIM ARASTIRMA HASTANESI | 1 | 0.109 |
| SAMUEL MERRITT COLL | 1 | 0.109 |
| SAN CAMILLO FORLANINI HOSP | 1 | 0.109 |
| SAN DIEGO STATE UNIV | 1 | 0.109 |
| SAN DIEGO VA MED CTR | 1 | 0.109 |
| SAN ELOY HOSP | 1 | 0.109 |
| SANFORD BURNHAM INST MED RES | 1 | 0.109 |
| SANLIURFA MEHMET AKIF INAN EDUC RES HOSP | 1 | 0.109 |
| SANOFI PASTEUR MSD | 1 | 0.109 |
| SAPPORO MED COLL | 1 | 0.109 |
| SCH PUBL HLTH | 1 | 0.109 |
| SCUOLA SUPER SANT ANNA | 1 | 0.109 |
| SE LOUISIANA VET HLTH CARE SYST | 1 | 0.109 |
| SEATTLE VET MED CTR | 1 | 0.109 |
| SELFCARE FIRST LLC | 1 | 0.109 |
| SENBOKU KUMIAI GEN HOSP | 1 | 0.109 |
| SEOUL NANUM PAIN CLIN | 1 | 0.109 |
| SEOUL NATL UNIV BUNDANG HOSP | 1 | 0.109 |
| SESCAM | 1 | 0.109 |
| SHAANXI UNIV CHINESE MED | 1 | 0.109 |
| SHANGHAI CHEMPARTNER CO LTD | 1 | 0.109 |
| SHANGHAI ELIXIRIA BIOTECH CO LTD | 1 | 0.109 |
| SHANGHAI JIAN QIAO UNIV | 1 | 0.109 |
| SHANGHAI UNIV MED HLTH SCI | 1 | 0.109 |
| SHANGHAI UNIV SPORT | 1 | 0.109 |
| SHANGHAI UNIV TRADIT CHINESE MED | 1 | 0.109 |
| SHEFFIELD HLTH SOCIAL CARE NHS FDN TRUST | 1 | 0.109 |
| SHENZHEN UNIV | 1 | 0.109 |
| SHIFA TAMEER E MILLAT UNIV | 1 | 0.109 |
| SHIN KONG WU HO MEM HOSP | 1 | 0.109 |
| SHIN KONG WU HO SU MEM HOSP | 1 | 0.109 |
| SHIRAZ UNIV MED SCI | 1 | 0.109 |
| SHOW CHWAN MEM HOSP | 1 | 0.109 |
| SIFA UNIV | 1 | 0.109 |
| SILMED SP ZOO | 1 | 0.109 |
| SINGAPORE GEN HOSP | 1 | 0.109 |
| SINGHEALTH POLYCLIN BUKIT MERAH | 1 | 0.109 |
| SIRIRAJ HOSP | 1 | 0.109 |
| SISLI HAMIDIYE ETFAL TRAINING RES HOSP | 1 | 0.109 |
| SISMANOGL GEN HOSP | 1 | 0.109 |
| SIVAS CUMHURIYET UNIV | 1 | 0.109 |
| SKIRBALL INST BIOMOL MED | 1 | 0.109 |
| SKOKIE HOSP | 1 | 0.109 |
| SOGANG UNIV | 1 | 0.109 |
| SOPHIA THERAPEUT LLC | 1 | 0.109 |
| SOUTH TEXAS VET HEATH CARE SYST | 1 | 0.109 |
| SOUTHERN MED UNIV | 1 | 0.109 |
| SOUTHERN TAIWAN UNIV SCI TECHNOL | 1 | 0.109 |
| SOUTHWEST UNIV | 1 | 0.109 |
| SPINE INTERVENT PROGNOST STUDY GRP LEIDEN | 1 | 0.109 |
| SPITAL LINTH | 1 | 0.109 |
| SRH HSCH GESUNDHEIT UNIV APPL HLTH SCI | 1 | 0.109 |
| SRH UNIV | 1 | 0.109 |
| SRH WALD KLINIKUM GERA | 1 | 0.109 |
| ST BARTHOLOMEWS ROYAL LONDON SCH MED DENT | 1 | 0.109 |
| ST BONIFACE GEN HOSP | 1 | 0.109 |
| ST JOSEPHS HEALTHCARE | 1 | 0.109 |
| ST JOSEPHS PARKWOOD HOSP | 1 | 0.109 |
| ST LOUIS HOSP | 1 | 0.109 |
| ST LOUIS UNIV | 1 | 0.109 |
| ST LUKES ROOSEVELT | 1 | 0.109 |
| STATCONSULT GMBH | 1 | 0.109 |
| STATE HOSP KONYA TURKEY | 1 | 0.109 |
| STATE HOSP NIGDE | 1 | 0.109 |
| STATE UNIV NEW JERSEY | 1 | 0.109 |
| STATE UNIV NOVI PAZAR | 1 | 0.109 |
| STN ZOOL A DOHRN | 1 | 0.109 |
| STOCKHOLM CTY COUNCIL | 1 | 0.109 |
| STONE LION VET HOSP | 1 | 0.109 |
| SUBEI PEOPLES HOSP JIANGSU PROV | 1 | 0.109 |
| SUBURBAN HOSP | 1 | 0.109 |
| SUNY HLTH SCI CTR | 1 | 0.109 |
| SWANSEA UNIV | 1 | 0.109 |
| SWEDISH MED CTR | 1 | 0.109 |
| SYDNEY CHILDRENS HOSP | 1 | 0.109 |
| SYDNEY MED SCH | 1 | 0.109 |
| SYDNEY SCH PUBL HLTH | 1 | 0.109 |
| TAICHUNG VET GEN HOSP | 1 | 0.109 |
| TAIZHOU PEOPLES HOSP | 1 | 0.109 |
| TAKARAZUKA UNIV MED HLTH CARE | 1 | 0.109 |
| TAMPERE UNIV HOSP | 1 | 0.109 |
| TAOYUAN ARMED FORCES GEN HOSP | 1 | 0.109 |
| TAWISHAN MED UNIV | 1 | 0.109 |
| TECHNION | 1 | 0.109 |
| TEXAS A M HLTH SCI CTR | 1 | 0.109 |
| TEXAS STATE UNIV SAN MARCOS | 1 | 0.109 |
| TEXAS TECH HLTH SCI CTR | 1 | 0.109 |
| TH KOLN | 1 | 0.109 |
| THERANEXUS | 1 | 0.109 |
| THRIASSIO GEN HOSP | 1 | 0.109 |
| TIANJIN INST ENVIRONM OCCUPAT MED | 1 | 0.109 |
| TOHO UNIV | 1 | 0.109 |
| TOKAI UNIV | 1 | 0.109 |
| TORAY INDUSTRIES LTD | 1 | 0.109 |
| TORREY PINES INST MOL STUDIES | 1 | 0.109 |
| TRAUMA RELATED NEURONAL DYSFUNCT TREND | 1 | 0.109 |
| TRIAL FORM SUPPORT SPAIN | 1 | 0.109 |
| TRIAL FORM SUPPORT TFS SPAIN | 1 | 0.109 |
| TRISERV GEN HOSP | 1 | 0.109 |
| TUFTS UNIV | 1 | 0.109 |
| TUNIS EL MANAR UNIV | 1 | 0.109 |
| TURKU UNIV HOSP | 1 | 0.109 |
| UBC SCI SOLUT | 1 | 0.109 |
| UCB LYON 1 | 1 | 0.109 |
| UCL HOSP NHS FDN TRUST | 1 | 0.109 |
| UCL INST NEUROL | 1 | 0.109 |
| UCL NATL HOSP NEUROL NEUROSURG | 1 | 0.109 |
| UCSD HNRC | 1 | 0.109 |
| UCSF | 1 | 0.109 |
| UIJEONGBU HOSP | 1 | 0.109 |
| UK COCHRANE CTR | 1 | 0.109 |
| ULSAN NATL INST SCI TECHNOL | 1 | 0.109 |
| ULSAN UNIV | 1 | 0.109 |
| ULSS8 | 1 | 0.109 |
| ULUDAG UNIV | 1 | 0.109 |
| UMASS MEM MED CTR | 1 | 0.109 |
| UMEA UNIV HOSP | 1 | 0.109 |
| UNC PROJECT MALAWI | 1 | 0.109 |
| UNICANCER | 1 | 0.109 |
| UNIFORMED SERV UNIV HLTH SCI | 1 | 0.109 |
| UNION COLL | 1 | 0.109 |
| UNION HOSP | 1 | 0.109 |
| UNITED BIOSOURCE CORP | 1 | 0.109 |
| UNIV AALBORG | 1 | 0.109 |
| UNIV ABERDEEN | 1 | 0.109 |
| UNIV ADELAIDE | 1 | 0.109 |
| UNIV ALABAMA | 1 | 0.109 |
| UNIV AMSTERDAM | 1 | 0.109 |
| UNIV ANCONA | 1 | 0.109 |
| UNIV ANTIOQUIA | 1 | 0.109 |
| UNIV ANTWERP HOSP | 1 | 0.109 |
| UNIV APPALACHIA | 1 | 0.109 |
| UNIV AQUILA | 1 | 0.109 |
| UNIV ARIZONA HLTH SCI | 1 | 0.109 |
| UNIV ATHENS | 1 | 0.109 |
| UNIV AUTONOMA MADRID | 1 | 0.109 |
| UNIV AUTONOMA NUEVO LEON | 1 | 0.109 |
| UNIV AUVERGNE | 1 | 0.109 |
| UNIV BALEAR ISLANDS | 1 | 0.109 |
| UNIV BASEL | 1 | 0.109 |
| UNIV BERN | 1 | 0.109 |
| UNIV BOLOGNA | 1 | 0.109 |
| UNIV BONN | 1 | 0.109 |
| UNIV BORDEAUX | 1 | 0.109 |
| UNIV BUCHAREST | 1 | 0.109 |
| UNIV CALABRIA | 1 | 0.109 |
| UNIV CALIF DAVIS | 1 | 0.109 |
| UNIV CALIF IRVINE | 1 | 0.109 |
| UNIV CAMPANIA | 1 | 0.109 |
| UNIV CAMPANIA L VANVITELLI | 1 | 0.109 |
| UNIV CAMPANIA NAPLES | 1 | 0.109 |
| UNIV CANTABRIA | 1 | 0.109 |
| UNIV CARLO BO | 1 | 0.109 |
| UNIV CHILE | 1 | 0.109 |
| UNIV CITY SAO PAULO | 1 | 0.109 |
| UNIV CITY SAO PAULO UNICID | 1 | 0.109 |
| UNIV CLAUDE BERNARD LYON 1 | 1 | 0.109 |
| UNIV CLIN TUBINGEN | 1 | 0.109 |
| UNIV CLIN VUK VRHOVAC | 1 | 0.109 |
| UNIV COLL DUBLIN | 1 | 0.109 |
| UNIV COLL LONDON HOSP NHS FDN TRUST | 1 | 0.109 |
| UNIV COMPLUTENSE MADRID | 1 | 0.109 |
| UNIV CYPRUS | 1 | 0.109 |
| UNIV DRESDEN | 1 | 0.109 |
| UNIV DSCHANG | 1 | 0.109 |
| UNIV DUISBURG ESSEN | 1 | 0.109 |
| UNIV ELECT SCI TECHNOL CHINA | 1 | 0.109 |
| UNIV ESTADO RIO DE JANEIRO | 1 | 0.109 |
| UNIV ESTADUAL CAMPINAS | 1 | 0.109 |
| UNIV ESTADUAL PAULISTA | 1 | 0.109 |
| UNIV FED BAHIA | 1 | 0.109 |
| UNIV FED CIENCIAS SAUDE PORTO ALEGRE | 1 | 0.109 |
| UNIV FED MINAS GERAIS | 1 | 0.109 |
| UNIV FED PAMPA | 1 | 0.109 |
| UNIV FED PARAIBA | 1 | 0.109 |
| UNIV FED PELOTAS | 1 | 0.109 |
| UNIV FED PELOTAS UFPEL | 1 | 0.109 |
| UNIV FED RIO DE JANEIRO | 1 | 0.109 |
| UNIV FED SAO PAULO | 1 | 0.109 |
| UNIV FLORENCE | 1 | 0.109 |
| UNIV FLORIDA | 1 | 0.109 |
| UNIV FREIBURG | 1 | 0.109 |
| UNIV FREIBURG KLINIKUM | 1 | 0.109 |
| UNIV FRIBOURG | 1 | 0.109 |
| UNIV GIESSEN | 1 | 0.109 |
| UNIV GRENOBLE ALPES | 1 | 0.109 |
| UNIV GRONINGEN | 1 | 0.109 |
| UNIV HALLE WITTENBERG | 1 | 0.109 |
| UNIV HAWAII MANOA | 1 | 0.109 |
| UNIV HOSP BALGRIST | 1 | 0.109 |
| UNIV HOSP BESANCON | 1 | 0.109 |
| UNIV HOSP BONN | 1 | 0.109 |
| UNIV HOSP BRNO | 1 | 0.109 |
| UNIV HOSP CHU | 1 | 0.109 |
| UNIV HOSP CTR SESTRE MILOSRDNICE | 1 | 0.109 |
| UNIV HOSP CTR ZAGREB | 1 | 0.109 |
| UNIV HOSP DUSSELDORF | 1 | 0.109 |
| UNIV HOSP FDN A GEMELLI | 1 | 0.109 |
| UNIV HOSP FREIBURG | 1 | 0.109 |
| UNIV HOSP GENEVA | 1 | 0.109 |
| UNIV HOSP LEICESTER NHS TRUST | 1 | 0.109 |
| UNIV HOSP LEUVEN | 1 | 0.109 |
| UNIV HOSP MANNHEIM | 1 | 0.109 |
| UNIV HOSP POITIERS | 1 | 0.109 |
| UNIV HOSP PUERTA DEL MAR | 1 | 0.109 |
| UNIV HOSP VALL HEBRON | 1 | 0.109 |
| UNIV INNSBRUCK | 1 | 0.109 |
| UNIV KANSAS | 1 | 0.109 |
| UNIV KLINIKUM DUSSELDORF | 1 | 0.109 |
| UNIV KRAGUJEVAC | 1 | 0.109 |
| UNIV LANCASTER | 1 | 0.109 |
| UNIV LEICESTER | 1 | 0.109 |
| UNIV LONDON ROYAL VET COLL | 1 | 0.109 |
| UNIV LOUISVILLE | 1 | 0.109 |
| UNIV LUND HOSP | 1 | 0.109 |
| UNIV MADISON WISCONSIN | 1 | 0.109 |
| UNIV MANITOBA | 1 | 0.109 |
| UNIV MAROUA | 1 | 0.109 |
| UNIV MASSACHUSETTS | 1 | 0.109 |
| UNIV MED | 1 | 0.109 |
| UNIV MED CTR | 1 | 0.109 |
| UNIV MED CTR GOTTINGEN | 1 | 0.109 |
| UNIV MED CTR HEIDELBERG | 1 | 0.109 |
| UNIV MED CTR TUBINGEN | 1 | 0.109 |
| UNIV MED GOTTINGEN | 1 | 0.109 |
| UNIV MED SCI | 1 | 0.109 |
| UNIV MELBOURNE | 1 | 0.109 |
| UNIV MILAN | 1 | 0.109 |
| UNIV MILANO BICOCCA | 1 | 0.109 |
| UNIV MISSISSIPPI | 1 | 0.109 |
| UNIV MISSOURI | 1 | 0.109 |
| UNIV MONTPELLIER | 1 | 0.109 |
| UNIV MUNSTER | 1 | 0.109 |
| UNIV MURCIA | 1 | 0.109 |
| UNIV N CAROLINA | 1 | 0.109 |
| UNIV NAPLES FEDERICO II | 1 | 0.109 |
| UNIV NEW ENGLAND | 1 | 0.109 |
| UNIV NEW S WALES | 1 | 0.109 |
| UNIV NEWCASTLE | 1 | 0.109 |
| UNIV NIJMEGEN HOSP | 1 | 0.109 |
| UNIV OCCUPAT ENVIRONM HLTH | 1 | 0.109 |
| UNIV ORTHOPAED HAND RECONSTRUCT MICROSURG CLUST | 1 | 0.109 |
| UNIV OSLO | 1 | 0.109 |
| UNIV OULU | 1 | 0.109 |
| UNIV PACIFIC | 1 | 0.109 |
| UNIV PADUA | 1 | 0.109 |
| UNIV PARIS 06 | 1 | 0.109 |
| UNIV PARIS 11 | 1 | 0.109 |
| UNIV PARIS DESCARTES PARIS DIDEROT | 1 | 0.109 |
| UNIV PARIS DIDERO | 1 | 0.109 |
| UNIV PARIS EST CRETEIL | 1 | 0.109 |
| UNIV PECS | 1 | 0.109 |
| UNIV PIEMONTE ORIENTALE | 1 | 0.109 |
| UNIV PORTO | 1 | 0.109 |
| UNIV PORTO ISPUP | 1 | 0.109 |
| UNIV PSYCHIAT HOSP AARHUS | 1 | 0.109 |
| UNIV PUERTO RICO | 1 | 0.109 |
| UNIV READING | 1 | 0.109 |
| UNIV REIMS | 1 | 0.109 |
| UNIV REY JUAN CARLOS I | 1 | 0.109 |
| UNIV S AUSTRALIA | 1 | 0.109 |
| UNIV S FLORIDA | 1 | 0.109 |
| UNIV SALENTO | 1 | 0.109 |
| UNIV SAN JORGE | 1 | 0.109 |
| UNIV SAN PABLO | 1 | 0.109 |
| UNIV SANTIAGO CHILE | 1 | 0.109 |
| UNIV SANTO TOMAS HOSP | 1 | 0.109 |
| UNIV SAO PAULO FMRP USP | 1 | 0.109 |
| UNIV SAPIENZA | 1 | 0.109 |
| UNIV SCH MED | 1 | 0.109 |
| UNIV SCHLESWIG HOLSTEIN | 1 | 0.109 |
| UNIV SHERBROOKE | 1 | 0.109 |
| UNIV SO CALIF | 1 | 0.109 |
| UNIV SO DENMARK | 1 | 0.109 |
| UNIV SOUTHERN SWITZERLAND | 1 | 0.109 |
| UNIV SPLIT | 1 | 0.109 |
| UNIV STOCKHOLM | 1 | 0.109 |
| UNIV SUL SANTA CATARINA UNISUL | 1 | 0.109 |
| UNIV TERAMO | 1 | 0.109 |
| UNIV TEXAS HLTH SCI CTR SAN ANTONIO | 1 | 0.109 |
| UNIV TEXAS SW MED CTR DALLAS | 1 | 0.109 |
| UNIV THESSALY | 1 | 0.109 |
| UNIV TURKU | 1 | 0.109 |
| UNIV ULM | 1 | 0.109 |
| UNIV ULSAN | 1 | 0.109 |
| UNIV UPPSALA HOSP | 1 | 0.109 |
| UNIV UTAH | 1 | 0.109 |
| UNIV VERACRUZANA | 1 | 0.109 |
| UNIV VERMONT | 1 | 0.109 |
| UNIV VERSAILLES ST QUENTIN YVELINES | 1 | 0.109 |
| UNIV VIENNA | 1 | 0.109 |
| UNIV VIRGINIA | 1 | 0.109 |
| UNIV WARWICK | 1 | 0.109 |
| UNIV WISCONSIN HOSP CLIN | 1 | 0.109 |
| UNIV WITWATERSRAND | 1 | 0.109 |
| UNIV YAMANASHI | 1 | 0.109 |
| UNIV YAOUNDE I | 1 | 0.109 |
| UNIV ZAGREB | 1 | 0.109 |
| UNIV ZARAGOZA | 1 | 0.109 |
| UNIV ZIMBABWE | 1 | 0.109 |
| UNIV ZURICH HOSP | 1 | 0.109 |
| UPR3212 CNRS | 1 | 0.109 |
| USAK EGITIM ARASRMA HASTANESI FIZ TEDAVI REHABI | 1 | 0.109 |
| USAK TRAINING RES HOSP | 1 | 0.109 |
| VA BOSTON HEALTHCARE SYST | 1 | 0.109 |
| VERSAILLES ST QUENTIN EN YVELINES UNIV | 1 | 0.109 |
| VET ADM HOSP | 1 | 0.109 |
| VICTOR BABES NATL INST PATHOL | 1 | 0.109 |
| VICTOR BABES UNIV MED PHARM | 1 | 0.109 |
| VIECURI MED CTR | 1 | 0.109 |
| VILLECRESNES MED HOSP | 1 | 0.109 |
| VIVEKANANDA INST MED SCI | 1 | 0.109 |
| WACO FAMILY HLTH CTR | 1 | 0.109 |
| WAKE FOREST UNIV | 1 | 0.109 |
| WAKE FOREST UNIV HLTH SCI | 1 | 0.109 |
| WALTER REED ARMY INST RES | 1 | 0.109 |
| WALTON CTR NEUROL NEUROSURG | 1 | 0.109 |
| WASHINGTON STATE UNIV | 1 | 0.109 |
| WEIHENSTEPHAN TRIESDORF UNIV APPL SCI | 1 | 0.109 |
| WELLMONT HLTH SYST | 1 | 0.109 |
| WESTERN UNIV | 1 | 0.109 |
| WESTMEAD HOSP | 1 | 0.109 |
| WILLAMETTE UNIV | 1 | 0.109 |
| WINGATE UNIV | 1 | 0.109 |
| WOUND CARE CTR NE WYOMING | 1 | 0.109 |
| WRAIR | 1 | 0.109 |
| WRIGHT STATE UNIV | 1 | 0.109 |
| WUHAN UNIV | 1 | 0.109 |
| XALUD THERAPEUT | 1 | 0.109 |
| XENOPORT INC | 1 | 0.109 |
| XIAN JIAOTONG UNIV | 1 | 0.109 |
| XINXIANG MED COLL | 1 | 0.109 |
| XINXIANG MED UNIV | 1 | 0.109 |
| XUZHOU MED COLL | 1 | 0.109 |
| YANTAI VOCAT SCH | 1 | 0.109 |
| YEDITEPE UNIV | 1 | 0.109 |
| YEUNGNAM UNIV | 1 | 0.109 |
| YUANLIN CHRISTIAN HOSP | 1 | 0.109 |
| ZABLOCKI VET ADM MED CTR | 1 | 0.109 |
| ZABLOCKI VET AFFAIRS MED CTR | 1 | 0.109 |
| ZANJAN UNIV MED SCI | 1 | 0.109 |
| ZB MED INFORMAT CTR LIFE SCI | 1 | 0.109 |
| ZENTRALKLIN BAD BERKA | 1 | 0.109 |
| ZHANGQIU HOSP TRADIT CHINESE MED | 1 | 0.109 |
| ZHEJIANG PROV PEOPLES HOSP | 1 | 0.109 |
| ZOUPING HOSP TCM | 1 | 0.109 |
| ZUNYI MED UNIV | 1 | 0.109 |
